# Supplementary material for: Characterising the Physiological Responses of Chinook Salmon (Oncorhynchus tshawytscha) Subjected to Heat and Oxygen Stress
Source: Biology (Basel). 2023 Oct 17;12(10):1342. doi: 10.3390/biology12101342 (PMC10604766; doi:10.3390/biology12101342)
Supplement: Supplementary file 1 [file biology-12-01342-s001.zip › biology-2497437-supplementary/E. Supplementary Figures no track changes.pdf]

Supplementary Figures

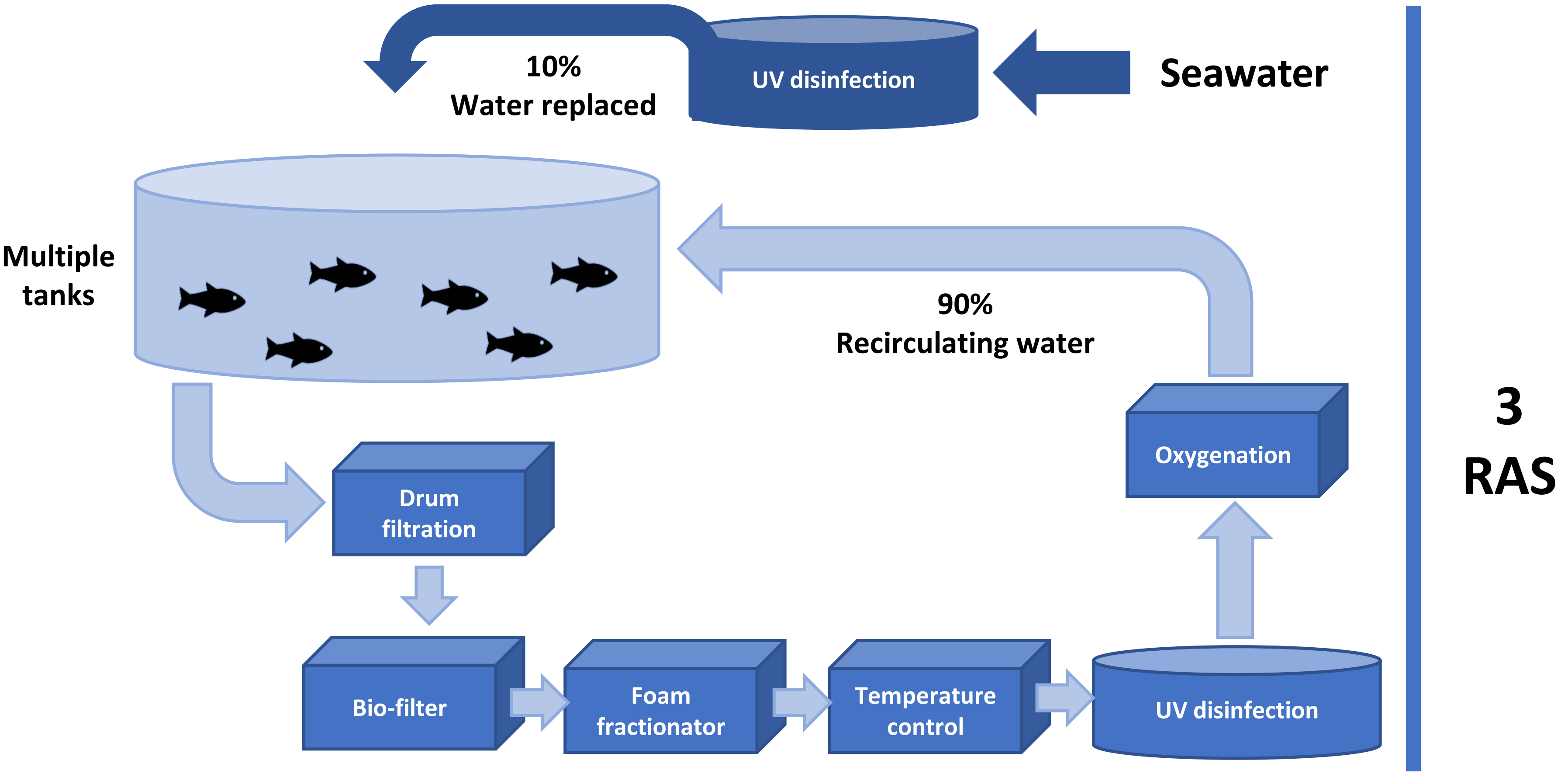

Figure S1: Recirculating aquaculture system (RAS) setup within the Finfish Research Centre (FRC), Cawthron Institute. For this investigation, three separate RAS systems, each consisting of 4-5 x 8000 L tanks were utilized.

ATGTCCGATAACGAAGGAGAgtaagtatatagaatattatctacattttaatttttttttttaagtacaacgatg

M S D N E G D

-----------------------------------210 bp-----------------------------------

aactctccactgttcacgttttcgtgtattattctttactcctcttattacctctgcagTTTTGATGATGGA

F D D G

GACTTTGATGACGCTGAAGAGGATGAGGGATTGGATGACCTGGAAAATGTTGAAGATgtgagtttgctacatatt

D F D D A E E D E G L D D L E N V E D

-----------------------------------210 bp-----------------------------------

tctttgcgctctctgtctgtttcttgttatcctgacctgttacgagGAGGACCAAGAGAATGTGAAGATCCTGCC

E D Q E N V K I L P

TGCAGGGGAGGGGTCGCAGGCTAACCAGAAGAGGATCACAACCCAATACATGACCAAATATGAAAGGGCCAGGGT

A G E G S Q A N Q K R I T T Q Y M T K Y E R A R V

GCTGGGGACACGTGCCCTCCAGATAGCgtgagtacacctcagcagtgttgcaatacgctaggaaagtgaaacctc

L G T R A L Q I A

-----------------------------------675 bp-----------------------------------

atctctgtcatgttgtcactattcataactgtctctgtgatatagGATGTGTGCCCCAGTCATGGTGGAGCTGGA

M C A P V M V E L E

GGGAGAGACAGACCCTCTGCAAATTGCCATGAAAGAGCTAAAgtaagctatgcttaatcttgttttttttctagt

G E T D P L Q I A M K E L K

-----------------------------------30 bp-----------------------------------

acttggagagtgcatgttactcttcagtatatgattgagtgtgtgaccgttacagGTGCAGGAAGATCCCCATCA

C R K I P I

TCATCCGGCGGTACCTTCCTGATGGCAGTTATGAAGATTGGGGCTGTGACGAGCTCATCATCACAGACTGA

I I R R Y L P D G S Y E D W G C D E L I I T D -

Figure S2: Compiled full-length Chinook salmon POLR2Fa genomic sequence taken from LG09 (GenBank accession No. NC_056437.1) with the cDNA sequence for POLR2Fa (GenBank accession no. OQ215307) highlighted and predicted amino acid sequence shown. The open reading frame is shown in upper-case letters and introns are in lowercase. Two conserved regions are highlighted, an acidic segment in the N-terminal region (dark grey) and a C-terminal region (light grey), essential for the enzymes activity. The RNA polymerases K / 14 to 18 Kd subunits signature is highlighted in red. The following conserved regions are boxed; Casein kinase II phosphorylation sites (orange), cAMP- and cGMP-dependent protein kinase phosphorylation site (pink) and Tyrosine kinase phosphorylation site 2 (purple).

```
ATGTCCGACAACGAAGGAGAgtaagtatataggatattgttaactaaaaaaaaatgaagtacaacaatgtgtgtc
M  S  D  N  E  G  D
-----------------------------------138 bp-----------------------------------
ctccacggttcatcttttagtgtattattctttacactttactcctcttgttacctctgcagTTTTGATGATGGA
                                                                F  D  D  G
GACTTTGATGACGCTGAAGAGGATGAAGGATTGGATGACCTAGAAAACGTTGAAGATgtgagtttgctacatatt
D  F  D  D  A  E  E  D  E  G  L  D  D  L  E  N  V  E  D
-----------------------------------198 bp-----------------------------------
ccaacctcttcctctttgcgctctctgtctgtttcttgttatcctgacctgttacgagGAGGACCAAGAGAATGT
                                                           E  D  Q  E  N  V
GAAGATCCTGCCTGCAGGGGAGGGGTCACAGGCCAACCAGAAGAGGATCACAACCCAATACATGACCAAATATGA
 K  I  L  P  A  G  E  G  S  Q  A  N  Q  K  R  I  T  T  Q  Y  M  T  K  Y  E
AAGAGCCAGGGTGCTGGGGACACGCGCCCTCCAGATAGCgtgagtacacctcagcagtgttgcaatacgctagga
 R  A  R  V  L  G  T  R  A  L  Q  I  A
-----------------------------------708 bp-----------------------------------
tgtctctgtgatatagCATGTGTGCCCCAGTCATGGTGGAGCTGGAGGGAGAGACAGACCCTCTGCAAATTGCCA
                  M  C  A  P  V  M  V  E  L  E  G  E  T  D  P  L  Q  I  A
TGAAAGAGCTAAAgtaagctatgcttaatcttgtttttttctagttcatgatggaaggagtctatacagtttga
M  K  E  L  K
-----------------------------------40 bp------------------------------------
tgtgtgaccgttacagGTGCAGGAAGATCCCCATCATCATCCGGCGGTACCTTCCTGATGGCAGTTATGAAGATT
                  C  R  K  I  P  I  I  I  R  R  Y  L  P  D  G  S  Y  E  D
GGGGCTGTGACGAGCTCATCATCACAGACTGA
W  G  C  D  E  L  I  I  T  D  -
```

Figure S3: Compiled full-length Chinook salmon POLR2Fb genomic sequence taken from LG27 (GenBank accession no. NC_056455.1) with the cDNA sequence for POLR2Fb (GenBank accession no. OQ215308) highlighted and predicted amino acid sequence shown. The open reading frame is shown in upper-case letters and introns are in lowercase. Two conserved regions are highlighted, an acidic segment in the N-terminal region (dark grey) and a C-terminal region (light grey), essential for the enzymes activity. The RNA polymerases K / 14 to 18 Kd subunits signature is highlighted in red. The following conserved regions are boxed; Casein kinase II phosphorylation sites (orange), cAMP- and cGMP-dependent protein kinase phosphorylation site (pink) and Tyrosine kinase phosphorylation site 2 (purple).

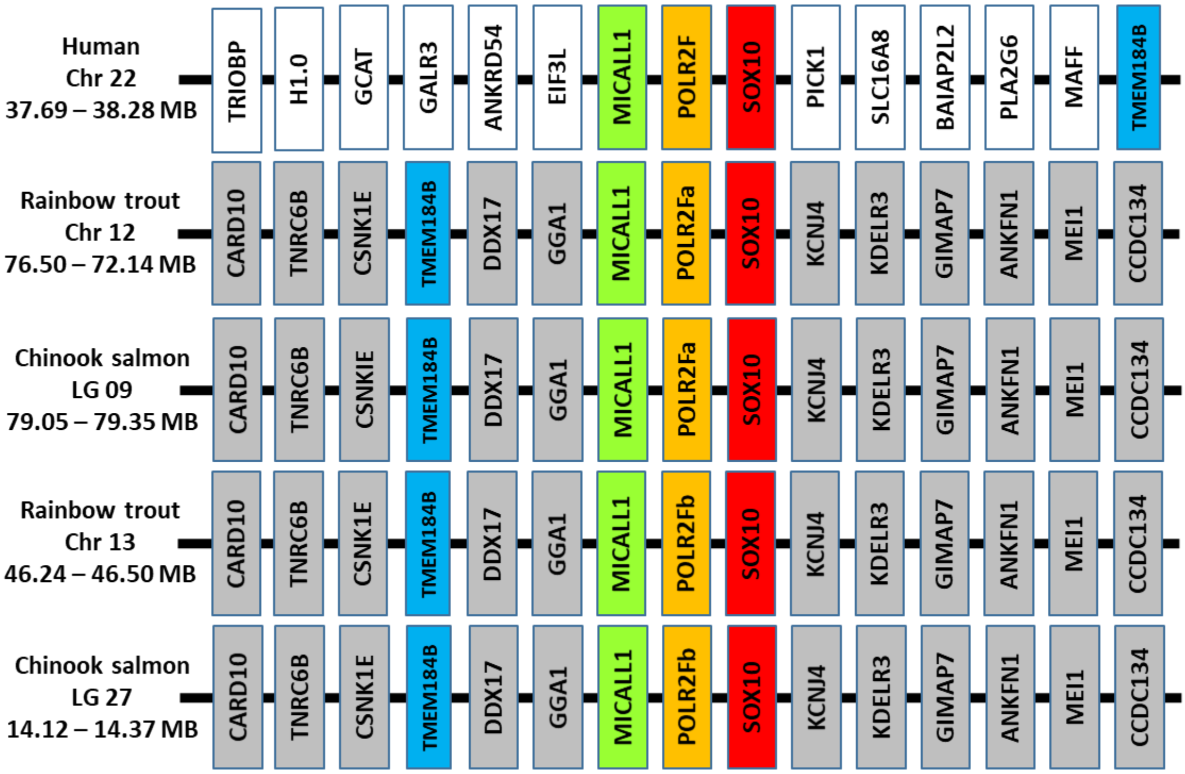

Figure S4: Synteny analysis of the locus containing the POLR2F genes from human (chromosome 22) and selected salmonid genomes. GenBank accession numbers of the salmonid genomes analysed: Rainbow trout chromosome 12 (GenBank accession No. CM046581.1), Chinook salmon LG09 (GenBank accession No. NC_056437.1), Rainbow trout chromosome 13 (GenBank accession No. CM046582.1), Chinook salmon LG27 (GenBank accession No. NC_056455.1). The chinook salmon and rainbow trout POLR2F genes showed conserved linkage of two human chromosome 22 genes: Molecules Interacting with CasL-like 1 (MICALL1) and SRY-Box Transcription Factor 10 (SOX10).

```
Human                   MSDNEDNFDGDDFDDVEEDEGLDDLENAEEEGQENVEILPSGERP  45
Green anole             MSDNEDNFDGDDFDDVEEDEGLDDLENAEEEGQENVEILPSGERQ..45
Chicken                 MSDNEDNFDGDDFDDVEEDEGLDDLENVEEEGQENVEILPSGERQ  45
Tropical clawed frog    MSDNEDNFDGDDFDDVEEDEGLDDLDNAEEEDQENVAILPAGEGQ  45
Rainbow trout a         MSDNEGDFDDGDFDDAEEDEGLDDLENVEDEDQENVKILPAGEGS  45
Rainbow trout b         MSDNEGDFDDGDFDDAEEDEGLDDLENVEDEDQENVKILPAGEGS  45
Chinook salmon a        MSDNEGDFDDGDFDDAEEDEGLDDLENVEDEDQENVKILPAGEGS  45
Chinook salmon b        MSDNEGDFDDGDFDDAEEDEGLDDLENVEDEDQENVKILPAGEGS  45
Small-spotted catshark  MSDNEDNFDGDDFDDADEDEGMDDLDNAEEEDRENVEILPAADGQ  45
Sea lamprey             MSDNEDNFDGDDF--AEDEEAMDDLDNAEEEGEERVEVLAAGDGT  43
                        *****.:**..**  .:::*.:***:*.*:*..*.*  :*.:.:

Human                   QANQKRITTPYMTKYERARVLGTRALQIAMCAPVMVELEGETDPL  95
Green anole             QANQKRITTPYMTKYERARVLGTRALQIAMCAPVMVELEGETDPL  95
Chicken                 AANQKRITTPYMTKYERARVLGTRALQIAMCAPVMVELEGETDPL  95
Tropical clawed frog    QANQKRITTNYMTKYERARVLGTRALQIAMCAPVMVELEGETDPL  95
Rainbow trout a         QANQKRITTQYMTKYERARVLGTRALQIAMCAPVMVELEGETDPL  95
Rainbow trout b         QANQKRITTQYMTKYERARVLGTRALQIAMCAPVMVELEGETDPL  95
Chinook salmon a        QANQKRITTQYMTKYERARVLGTRALQIAMCAPVMVELEGETDPL  95
Chinook salmon b        QANQKRITTQYMTKYERARVLGTRALQIAMCAPVMVELEGETDPL  95
Small-spotted catshark  QANQKRITTSYMTKYERARVLGTRALQIAMCAPVMVELEGETDPL  95
Sea lamprey             PGAAKRITTPYMTKYERARVLGTRALQIAMCAPVMVELEGETDPL  93
                         .  ***** ****************************************

Human                   LIAMKELKARKIPIIIRRYLPDGSYEDWGVDELIITD  127
Green anole             LIAMKELKARKIPIIIRRYLPDGSYEDWGVDELIITD  127
Chicken                 LIAMKELKARKIPIIIRRYLPDGSYEDWGVDELIITD  127
Tropical clawed frog    LIAMKELKARKIPIIIRRYLPDGSYEDWGVDELIITD  127
Rainbow trout a         QIAMKELKCRKIPIIIRRYLPDGSYEDWGCDELIITD  127
Rainbow trout b         QIAMKELKCRKIPIIIRRYLPDGSYEDWGCDELIITD  127
Chinook salmon a        QIAMKELKCRKIPIIIRRYLPDGSYEDWGCDELIITD  127
Chinook salmon b        QIAMKELKCRKIPIIIRRYLPDGSYEDWGCDELIITD  127
Small-spotted catshark  QIAMKELKARKIPIIIRRYLPDGSYEDWGVDELIITD  127
Sea lamprey             QIAIKELKARKIPIIIRRYLPDGSYEDWGVDELIVTD  125
                         **:****.********************* ****:**
```

Figure S5: Multiple alignment of the predicted Chinook salmon POLR2F translation with known vertebrate POLR2F molecules. Identical (*) and similar (: or .) residues identified by the Clustal omega program are indicated. Two conserved regions are highlighted, an acidic segment in the N-terminal region (dark grey) and a C-terminal region (light grey), essential for the enzymes activity. The RNA polymerases K / 14 to 18 Kd subunits signature is highlighted in red. The following conserved regions are boxed; Casein kinase II phosphorylation sites (orange), cAMP- and cGMP-dependent protein kinase phosphorylation site (pink) and Tyrosine kinase phosphorylation site 2 (purple). GenBank accession numbers of the POLR2F genes are: Human, NP_068809.1; Green anole, XP_003227193.1; Chicken, NP_990065.1; Tropical clawed frog, NP_001006888.1; Rainbow trout POLR2Fa, Chr12 predicted; Rainbow trout POLR2Fb, Chr13 predicted; Chinook salmon POLR2Fa, OQ215307; Chinook salmon POLR2Fb, OQ215308; Small-spotted catshark, XP_038639773.1; Sea lamprey, XP_032832117.1.

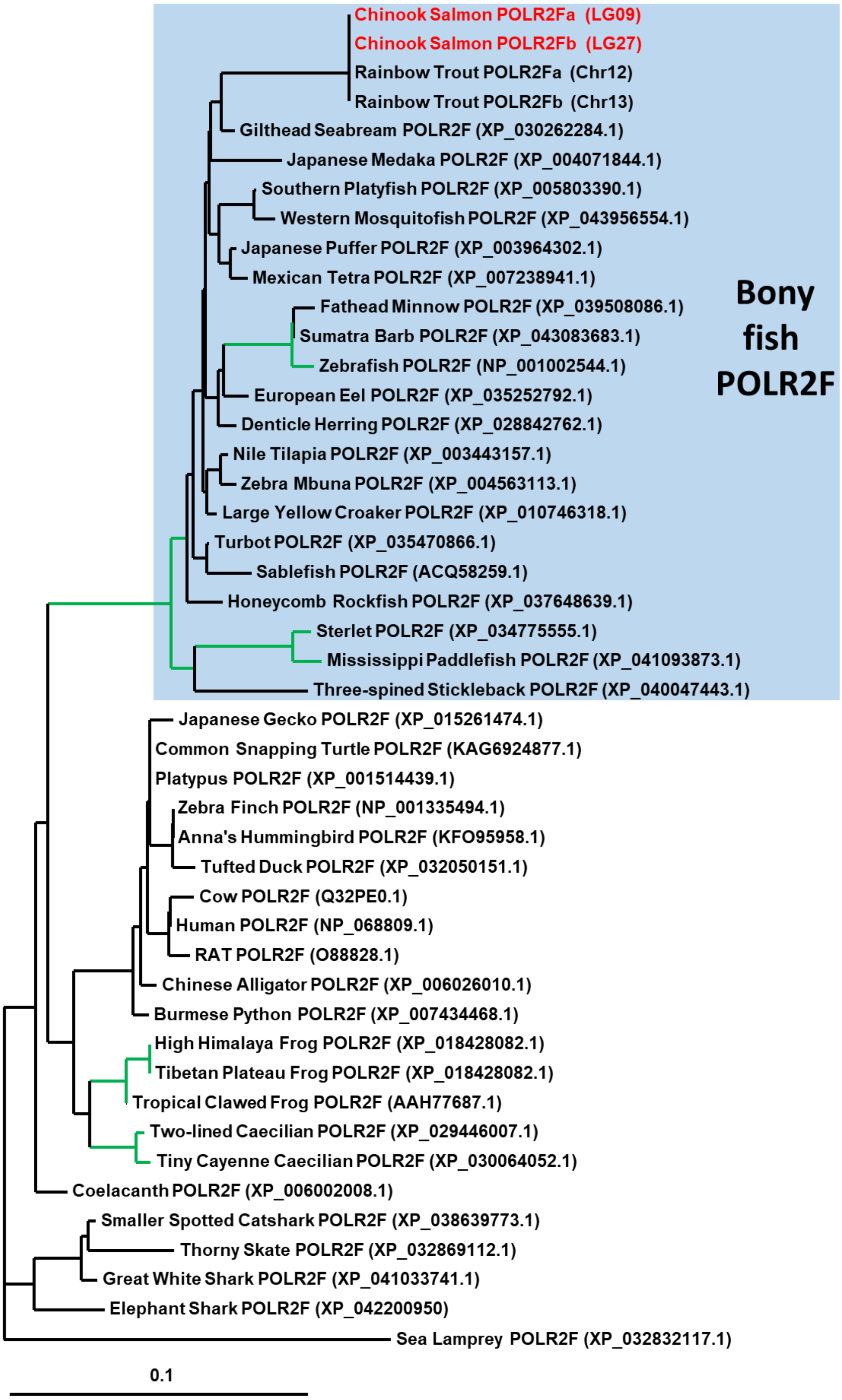

Figure S6: Unrooted phylogenetic tree showing the relationship between the Chinook salmon POLR2Fa and POLR2Fb amino acid sequences for the full-length molecule with other known selected vertebrate POLR2F sequences. This tree was constructed by the 'neighbour-joining' method using Clustal omega and iTOL v6. The tree was bootstrapped 10,000 times and branches supported >75% are highlighted green. The GenBank accession numbers for each organism are shown in brackets.

```
POLR2Fa     ATGTCCGACAACGAAGGAGATTTTGATGATGGAGACTTTGATGACGCTGAAGAGGATGAG   60
POLR2Fb     ATGTCCGACAACGAAGGAGATTTTGATGATGGAGACTTTGATGACGCTGAAGAGGATGAA   60
            ************************************************************

POLR2Fa     GGATTGGATGACCTGGAAAATGTTGAAGATGAGGACCAAGAGAATGTGAAGATCCTGCCT   120
POLR2Fb     GGATTGGATGACCTAGAAAACGTTGAAGATGAGGACCAAGAGAATGTGAAGATCCTGCCT   120
            ************** ***** ***************************************

POLR2Fa     GCAGGGGAGGGGTCGCAGGCTAACCAGAAGAGGATCACAACCCAATACATGACCAAATAT   180
POLR2Fb     GCAGGGGAGGGGTCACAGGCCAACCAGAAGAGGATCACAACCCAATACATGACCAAATAT   180
            ************** ***** ***************************************

POLR2Fa     GAAAGGGCCAGGGTGCTGGGGACACGTGCCCTCCAGATAGCGATGTGTGCCCCAGTCATG   240
POLR2Fb     GAAAGAGCCAGGGTGCTGGGGACACGCGCCCTCCAGATAGCCATGTGTGCCCCAGTCATG   240
            ***** ******************** ************** ******************

POLR2Fa     GTGGAGCTGGAGGGAGAGACAGACCCTCTGCAAATTGCCATGAAAGAGCTAAAGTGCAGG   300
POLR2Fb     GTGGAGCTGGAGGGAGAGACAGACCCTCTGCAAATTGCCATGAAAGAGCTAAAGTGCAGG   300
            ************************************************************

POLR2Fa     AAGATCCCCATCATCATCCGGCGGTACCTTCCTGATGGCAGTTATGAAGATTGGGGCTGT   360
POLR2Fb     AAGATCCCCATCATCATCCGGCGGTACCTTCCTGATGGCAGTTATGAAGATTGGGGCTGT   360
            ************************************************************

POLR2Fa     GACGAGCTCATCATCACAGACTGA   384
POLR2Fb     GACGAGCTCATCATCACAGACTGA   384
            ************************
```

Figure S7: Nucleotide alignment of Chinook salmon POLR2Fa (GenBank accession no. OQ215307) and POLR2Fb (GenBank accession no. OQ215308) cDNA sequences. Exons are highlighted with a different colour. Identical (*) nucleotides identified by the Clustal omega program are indicated. Positions where the forward and reverse primers were designed are boxed.

```
ATGgtaggtacagtttatgaaaacatcgttaactgacttgataatctgagtccatcctcacttttacagtagcac
 M
------------------------------------637 bp------------------------------------
gtatttacctgcaccatctattcccagGGAGTTGACATCCGACACAACAAGGACCGTAAGGTGCACAGAAAGGAG
                            G  V  D  I  R  H  N  K  D  R  K  V  H  R  K  E
CCCAAGAGTCAGGATATCTACCTGAGGCTCCTGGTCAAGgtaagtgtcgctacctttatacattaaggcattgtc
 P  K  S  Q  D  I  Y  L  R  L  L  V  K
------------------------------------148 bp------------------------------------
taaatgtttatgttctcttccccaaagCTGTACAGATTCCTGGCCCGTCGCTCCACTGCTCCCTTCAACAAGGTG
                            L  Y  R  F  L  A  R  R  S  T  A  P  F  N  K  V
GTCCTCAGGAGGCTCTTCATGAGCAGGACCCACAGGCCTCCGATATCAGTGTCCCGCATGgtgagttacatcaat
 V  L  R  R  L  F  M  S  R  T  H  R  P  P  I  S  V  S  R  M
-----------------------------------5790 bp------------------------------------
ctcttctgaactatttgccgtccttttcagATCCGTAAGATGAAACTGCCTGGACGTGAGAACAGAACCGCAGTT
                               I  R  K  M  K  L  P  G  R  E  N  R  T  A  V
GTCGTGGGAACCGTCACTGATGATGTCAGAATTCAGGATATCCCCAAGCTCAAGgtaaaggttgttttaacttac
 V  V  G  T  V  T  D  D  V  R  I  Q  D  I  P  K  L  K
------------------------------------310 bp------------------------------------
tctgttctcccttccctcagGTGTGCGCTCTGAAGGTGACTGACGGCGCTCGCCGCAGGATCCTGAAAGCCGGAG
                     V  C  A  L  K  V  T  D  G  A  R  R  R  I  L  K  A  G
GCCAGGTCATGACCTTTGACCAGCTGGCTCTGGCTGCCCCCAAAGGACAGGGCACCGTGCTGCTGTCAGgtaaga
G  Q  V  M  T  F  D  Q  L  A  L  A  A  P  K  G  Q  G  T  V  L  L  S
------------------------------------305 bp------------------------------------
agGACCCCGCAAGGGCAGAGAGGTGTACAGGCATTTTGGAAAAGCCTGTGGAACCCCCCACAGTCACACCAAgtg
  G  P  R  K  G  R  E  V  Y  R  H  F  G  K  A  C  G  T  P  H  S  H  T  K
-----------------------------------1870 bp------------------------------------
ttctctccctcagGCCCTACATTCGTTCCAAGGGCAGGAAGTTTGAACGTGCTCGTGGGCGCAGATCCAGCCGTG
                 P  Y  I  R  S  K  G  R  K  F  E  R  A  R  G  R  R  S  S  R
GATACAAGGCCTAA
G  Y  K  A  -
```

Figure S8: Compiled full-length Chinook salmon RPL18a genomic sequence taken from LG23 (GenBank accession No. NC_056451.1) with the cDNA sequence for RPL18a (GenBank accession no. OQ215309) highlighted and predicted amino acid sequence shown. The open reading frame is shown in upper-case letters and introns are in lowercase. The ribosomal protein L18e signature is highlighted in red. The protein kinase C phosphorylation sites are underlined. The following conserved regions are boxed; cAMP- and cGMP-dependent protein kinase phosphorylation site (pink), Casein kinase II phosphorylation sites (orange) and N-myristoylation site (green). The following conserved regions are highlighted; N-glycosylation site (light grey) and amidation site (dark grey).

```
ATGgtaggtacagattctgaaaacatcgttaatgctccgacttgataatctaagtccatcctcacttttacagta
 M
----------------------------------673 bp----------------------------------
atttacctgcaccatctattcacagGGAGTCGACATCCGACACAACAAGGACCGTAAGGTGCACCGAAAGGAGCC
                          G  V  D  I  R  H  N  K  D  R  K  V  H  R  K  E  P
CAAGAGTCAGGATATCTACCTGAGGCTCCTGGTCAAGgtaagtgtcacaacctttatacattgattcaagttaac
  K  S  Q  D  I  Y  L  R  L  L  V  K
----------------------------------162 bp----------------------------------
aaatgtttatgttctcttctccaaagCTGTACAGATTCCTGGCCCGTCGCTCCACTGCTCCCTTCAACAAGGTGG
                           L  Y  R  F  L  A  R  R  S  T  A  P  F  N  K  V
TCCTCAGGAGGCTCTTCATGAGCAGGACCCACAGGCCTCCGATATCAGTGTCCCGCATGgtgagttacatcaatc
V  L  R  R  L  F  M  S  R  T  H  R  P  P  I  S  V  S  R  M
---------------------------------3433 bp----------------------------------
ctcgtctgaactatttgacgtcctcttcagATCCGTAAGATGAAACTACCTGGTCGTGAGAACCGAACCGCAGTT
                               I  R  K  M  K  L  P  G  R  E  N  R  T  A  V
GTCGTGGGAACCGTCACTGATGATGTCAGAATTCAGGATATCCCCAAGCTCAAGgtaaaggttgttttaacttac
 V  V  G  T  V  T  D  D  V  R  I  Q  D  I  P  K  L  K
----------------------------------321 bp----------------------------------
ctctgttctcccttcccttagGTGTGCGCTCTGAAGGTGACTGACGGCGCTCGCCGCAGGATCCTGAAGGCCGGA
                       V  C  A  L  K  V  T  D  G  A  R  R  R  I  L  K  A  G
GGTCAGGTCATGACCTTTGACCAGCTGGCTCTGGCTGCCCCCAAAGGACAGGGCACCGTGCTGCTGTCAGgtaag
 G  Q  V  M  T  F  D  Q  L  A  L  A  A  P  K  G  Q  G  T  V  L  L  S
----------------------------------307 bp----------------------------------
agGACCCCGTAAGGGCAGAGAGGTGTACAGGCATTTTGGAAAAGCCTGTGGAACCCCCCACAGCCACACCAAgtg
  G  P  R  K  G  R  E  V  Y  R  H  F  G  K  A  C  G  T  P  H  S  H  T  K
---------------------------------1672 bp----------------------------------
ttctctccctcagGCCCTACATTCGCTCCAAGGGCAGGAAGTTTGAACGTGCTCGTGGGCGCAGATCCAGCCGTG
                P  Y  I  R  S  K  G  R  K  F  E  R  A  R  G  R  R  S  S  R
GATACAAGGCCTAA
G  Y  K  A  -
```

Figure S9: Compiled full-length chinook salmon RPL18b genomic sequence taken from LG03 (GenBank accession no. NC_056431.1) with the cDNA sequence for RPL18b (GenBank accession no. OQ215310) highlighted and predicted amino acid sequence shown. The open reading frame is shown in upper-case letters and introns are in lowercase. The ribosomal protein L18e signature is highlighted in red. The protein kinase C phosphorylation sites are underlined. The following conserved regions are boxed; cAMP- and cGMP-dependent protein kinase phosphorylation site (pink), Casein kinase II phosphorylation sites (orange) and N-myristoylation site (green). The following conserved regions are highlighted; N-glycosylation site (light grey) and amidation site (dark grey).

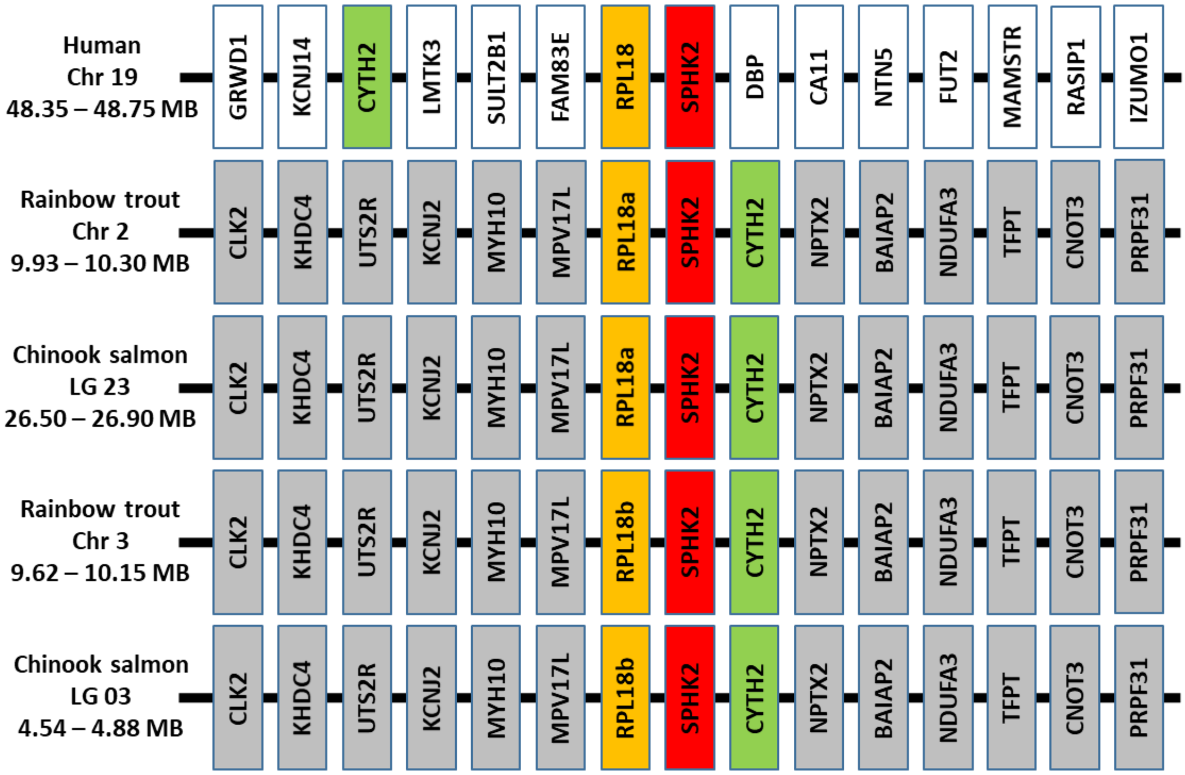

Figure S10: Synteny analysis of the locus containing the RPL18 genes from human (chromosome 19) and selected salmonid genomes. GenBank accession numbers of the salmonid genomes analysed: Rainbow trout chromosome 2 (GenBank accession No. CM046571.1), Chinook salmon LG03 (GenBank accession No. NC_056431.1), Rainbow trout chromosome 3 (GenBank accession No. CM046572.1), Chinook salmon LG23 (GenBank accession no. NC_056451.1). The Chinook salmon and rainbow trout RPL18 genes showed conserved linkage with one human chromosome 19 gene: Sphingosine Kinase 2 (SPHK2).

```
Human                 MGVDIRHNKDRKVRRKEPKSQDIYLRLLVKLYRFLARRTNSTFNQVVLKR  50
Ground Tit            MGIDIRHDKDRKVRRKEPKSQDIYLRLLVKLYRFLARRTNAPFNRVVLKR  50
Painted turtle        MGVDIRHNKDRKVRRTEPKSQDIYLRLLVKLYRFLARRTSSRFNKVVLKR  50
African clawed frog   MGIDIRHNKDRKVRRKEPKSQDIYLRLLVKLYRFLARRTNSSFNRVVLKR  50
Chinook salmon a      MGVDIRHNKDRKVHRKEPKSQDIYLRLLVKLYRFLARRSTAPFNKVVLRR  50
Chinook salmon b      MGVDIRHNKDRKVHRKEPKSQDIYLRLLVKLYRFLARRSTAPFNKVVLRR  50
Rainbow trout a       MGVDIRHNKDRKVHRKEPKSQDIYLRLLVKLYRFLARRSTAPFNKVVLRR  50
Rainbow trout b       MGVDIRHNKDRKVHRKEPKSQDIYLRLLVKLYRFLARRSTAPFNKVVLRR  50
Nurse shark           MGVDIRHNKDRKVRRKEPKSQDIYLRLLVKLYRFLARRSNAPFNKVVLRR  50
Sea lamprey           MGIDIRHNKDRKVRRTEPRSEDIYLRLLVKLYRFLARRTSSPFNKVVLKR  50
                      **:****:*****:*.**:*:*****************:.: **:***:*

Human                 LFMSRTNRPPLSLSRMIRKMKLPGRENKTAVVVGTITDDVRVQEVPKLKV  100
Ground tit            LFMSRSNRPPLALSRLIRMMRKPGRADKTAVVVGTVTDDVRIQNVPKLKV  100
Painted turtle        LFMSRTNRPPLSLSRMIRKMKLPGRDNKTAVVVGTVTDDIRIHDIPKLKV  100
African clawed frog   LFMSRTNRPPLSMSRLIRKMKLQGRENKTAVVVGYITDDVRIHDIPKLKV  100
Chinook salmon a      LFMSRTHRPPISVSRMIRKMKLPGRENRTAVVVGTVTDDVRIQDIPKLKV  100
Chinook salmon b      LFMSRTHRPPISVSRMIRKMKLPGRENRTAVVVGTVTDDVRIQDIPKLKV  100
Rainbow trout a       LFMSRTHRPPISVSRMIRKMKLPGRENRTAVVVGTVTDDVRIQDIPKLKV  100
Rainbow trout b       LFMSRTHRPPISVSRMIRKMKLPGRENRTAVVVGTVTDDVRIQDIPKLKV  100
Nurse shark           FFMSRTNRPPLSLARLIRKMKLPGREGKTAVIVGTVTDDVRVWEVPKLKV  100
Sea lamprey           LFMSRTNRPPISIARLVRKMKMGGREGKVAVVIGTITDDPRIFTIPKIKV  100
                      :****::***::::*::* *:  ** .:.**::* :*** *:  :**:**

Human                 CALRVTSRARSRILRAGGKILTFDQLALDSPKGCGTVLLSGPRKGREVYR  150
Ground tit            CALRVTRGARSRILRAGGSILTLDQLAMATPKGKGTVLLSGPRKAREVYR  150
Painted turtle        CALRVTQGARSRILKAGGQIMTFDQLAMAAPKGQGTVLLSGPRKGREVYR  150
African clawed frog   CALKITSGDRSRILKSGGQIMTFDQLALAAPKGQNTVLLSGPRKAREVYR  150
Chinook salmon a      CALKVTDGARRRILKAGGQVMTFDQLALAAPKGQGTVLLSGPRKGREVYR  150
Chinook salmon b      CALKVTDGARRRILKAGGQVMTFDQLALAAPKGQGTVLLSGPRKGREVYR  150
Rainbow trout a       CALKVTDGARRRILKAGGQVMTFDQLALAAPKGQGTVLLSGPRKGREVYR  150
Rainbow trout b       CALKVTDGARRRILKAGGQVMTFDQLALAAPKGQGTVLLSGPRKGREVYR  150
Nurse shark           CALRITDGARHRILKAGGQIMTFDQLALASPKGQGTVLLSGPRHGREVYR  150
Sea lamprey           CALRVTAKARDRILRAGGEVLTLDQLALLSPRGQNTVLLSGPRKGREAYR  150
                      ***::*   * ***::**.::*:****: :*:* .********:.**.**

Human                 HFGKAPGTPHSHTKPYVRSKGRKFERARGRRASRGYKN  188
Ground tit            HFGKAPGTPHSHTKPYVRSKGPKFERARGRRASRGYKN  188
Painted turtle        HFGKAPGTPHSHTKPYVRSKGRKFERARGRRASRAYKN  188
African clawed frog   HFGKAPGTPHSRTKPYVLSKGRKFERARGRRASRGYKN  188
Chinook salmon a      HFGKACGTPHSHTKPYIRSKGRKFERARGRRSSRGYKA  188
Chinook salmon b      HFGKACGTPHSHTKPYIRSKGRKFERARGRRSSRGYKA  188
Rainbow trout a       HFGKACGTPHSHTKPYIRSKGRKFERARGRRSSRGYKA  188
Rainbow trout b       HFGKACGTPHSHTKPYIRSKGRKFERARGRRSSRGYKA  188
Nurse shark           HFGPAPGTPHSHTKPYIQSKGRKFERARGRRASCGYKN  188
Sea lamprey           HFGPAPGVPHSHTKPYVRSKGRKFERARGRRASRGYKN  188
                      *** * *.***:****: *** *********:* .**
```

Figure S11: Multiple alignment of the predicted Chinook salmon RPL18a and RPL18b translation with known vertebrate RPL18 molecules. Identical (*) and similar (: or .) residues identified by the Clustal omega program are indicated. The ribosomal protein L18e signature is highlighted in red. The protein kinase C phosphorylation sites are underlined. The following conserved regions are boxed; cAMP- and cGMP-dependent protein kinase phosphorylation site (pink), Casein kinase II phosphorylation sites (orange) and N-myristoylation site (green). The following conserved regions are highlighted; N-glycosylation site (light grey) and amidation site (dark grey). GenBank accession numbers of the RPL18 genes are: Human RPL18, Q07020.2; Painted turtle RPL18, XP_005312106.1; Ground tit RPL18, XP_005534137.1; African clawed frog, P02412.4; Rainbow trout RPL18a, Chr2 predicted; Rainbow trout RPL18a, Chr3 predicted; Chinook salmon RPL18a, OQ215309; Chinook salmon RPL18b, OQ215310; Nurse shark RPL18, GIWU01078051.1; Sea lamprey RPL18, XP_032836522.1

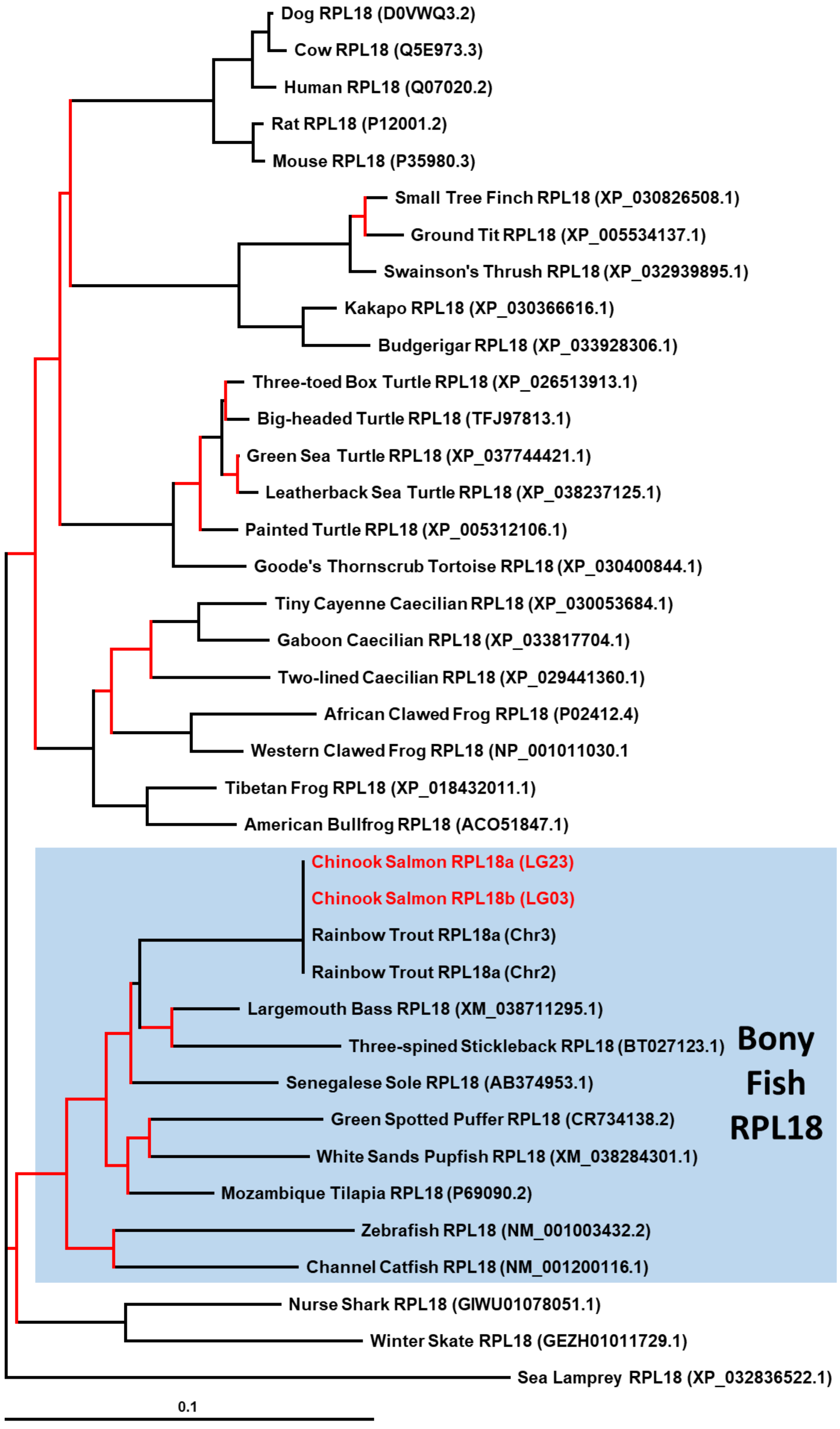

Figure S12: Unrooted phylogenetic tree showing the relationship between the Chinook salmon RPL18a and RPL18b amino acid sequences for the full-length molecule with other known selected vertebrate RPL18 sequences. This tree was constructed by the 'neighbour-joining' method using the Clustal omega and iTOL v6 packages. The tree was bootstrapped 10,000 times and branches supported <75% are highlighted red. The GenBank accession numbers for each organism are shown in brackets.

```
RPL18a      ATGGGAGTTGACATCCGACACAACAAGGACCGTAAGGTGCACAGAAAGGAGCCCAAGAGT   60
RPL18b      ATGGGAGTCGACATCCGACACAACAAGGACCGTAAGGTGCACCGAAAGGAGCCCAAGAGT   60
            ******** ********************************* *****************

RPL18a      CAGGATATCTACCTGAGGCTCCTGGTCAAGCTGTACAGATTCCTGGCCCGTCGCTCCACT   120
RPL18b      CAGGATATCTACCTGAGGCTCCTGGTCAAGCTGTACAGATTCCTGGCCCGTCGCTCCACT   120
            ************************************************************

RPL18a      GCTCCCTTCAACAAGGTGGTCCTCAGGAGGCTCTTCATGAGCAGGACCCACAGGCCTCCG   180
RPL18b      GCTCCCTTCAACAAGGTGGTCCTCAGGAGGCTCTTCATGAGCAGGACCCACAGGCCTCCG   180
            ************************************************************

RPL18a      ATATCAGTGTCCCGCATGATCCGTAAGATGAAACTGCCTGGACGTGAGAACAGAACCGCA   240
RPL18b      ATATCAGTGTCCCGCATGATCCGTAAGATGAAACTACCTGGTCGTGAGAACCGAACCGCA   240
            *********************************** ***** ********* ********

RPL18a      GTTGTCGTGGGAACCGTCACTGATGATGTCAGAATTCAGGATATCCCCAAGCTCAAGGTG   300
RPL18b      GTTGTCGTGGGAACCGTCACTGATGATGTCAGAATTCAGGATATCCCCAAGCTCAAGGTG   300
            ************************************************************

RPL18a      TGCGCTCTGAAGGTGACTGACGGCGCTCGCCGCAGGATCCTGAAAGCCGGAGGCCAGGTC   360
RPL18b      TGCGCTCTGAAGGTGACTGACGGCGCTCGCCGCAGGATCCTGAAGGCCGGAGGTCAGGTC   360
            ******************************************** ******** ******

RPL18a      ATGACCTTTGACCAGCTGGCTCTGGCTGCCCCCAAAGGACAGGGCACCGTGCTGCTGTCA   420
RPL18b      ATGACCTTTGACCAGCTGGCTCTGGCTGCCCCCAAAGGACAGGGCACCGTGCTGCTGTCA   420
            ************************************************************

RPL18a      GGACCCCGCAAGGGCAGAGAGGTGTACAGGCATTTTGGAAAAGCCTGTGGAACCCCCCAC   480
RPL18b      GGACCCCGTAAGGGCAGAGAGGTGTACAGGCATTTTGGAAAAGCCTGTGGAACCCCCCAC   480
            ******** ***************************************************

RPL18a      AGTCACACCAAGCCCTACATTCGTTCCAAGGGCAGGAAGTTTGAACGTGCTCGTGGGCGC   540
RPL18b      AGCCACACCAAGCCCTACATTCGCTCCAAGGGCAGGAAGTTTGAACGTGCTCGTGGGCGC   540
            ** ******************** ************************************

RPL18a      AGATCCAGCCGTGGATACAAGGCCTAA      567
RPL18b      AGATCCAGCCGTGGATACAAGGCCTAA      567
            ***************************
```

Figure S13: Nucleotide alignment of Chinook salmon RPL18a (GenBank accession no. OQ215309) and RPL18b (GenBank accession no. OQ215310) cDNA sequences. Exons are highlighted with a different colour. Identical (*) nucleotides identified by the Clustal omega program are indicated. Positions where the forward and reverse primers are found are boxed.

```
ATGGCAATGAAGGCTGTTTGCGTGCTCAAAGGCACCGGTGAAGTTACCGGGACCGTATTCTTTGAGCAGGAGgta
 M  A  M  K  A  V  C  V  L  K  G  T  G  E  V  T  G  T  V  F  F  E  Q  E
--------------------------------2011 bp-------------------------------------
cttgtctgtctgtagGGTGTCGATGGTCCAGTGAAGCTGATTGGGGTGATCTCTGGTCTGGCCCCGGGGAAACAT
                G  V  D  G  P  V  K  L  I  G  V  I  S  G  L  A  P  G  K  H
GGCTTCCACGTCCATGCTTTTGGAGACAACACCAACGGCTGTATGAGTGCCGGACCCCACTTTAACCCCCACAAC
 G  F  H  V  H  A  F  G  D  N  T  N  G  C  M  S  A  G  P  H  F  N  P  H  N
AAGACCCACGGAGGACCCACTGATGCTGTTCGgttagtctggcccccatgactgaaggagttaggcctgatagac
 K  T  H  G  G  P  T  D  A  V  R
--------------------------------1973 bp-------------------------------------
tgttgctagGCACGTAGGGGACCTTGGCAACGTGACTGCAGGAGCTGACAATGTGGCTAAGATCAACATCCAGGA
           H  V  G  D  L  G  N  V  T  A  G  A  D  N  V  A  K  I  N  I  Q  D
TGAGATGTTGACTCTCACTGGACCCAACTCTATCATCGGCAGGACCATGGTGgtaagactcatttttcagaattc
  E  M  L  T  L  T  G  P  N  S  I  I  G  R  T  M  V
--------------------------------102 bp--------------------------------------
tccccctttttatcattagATCCATGAGAAGGCTGATGATCTGGGAAAAGGAGGCAACGAGGAGAGTCTGAAGACT
                    I  H  E  K  A  D  D  L  G  K  G  G  N  E  E  S  L  K  T
GGCAACGCTGGCAGTCGGCAGGCCTGTGGCGTTATTGGCATTGCCCAGTAA
 G  N  A  G  S  R  Q  A  C  G  V  I  G  I  A  Q  -
```

Figure S14: Compiled full-length Chinook salmon SOD1a genomic sequence taken from LG33 (GenBank accession No. NC_056461.1) with the cDNA sequence for SOD1a (GenBank accession no. OP760294) highlighted and predicted amino acid sequences shown. The open reading frame is shown in upper-case letters and introns are in lowercase. Within the amino acid sequence, the two Cu/Zn SOD family signatures are underlined (_) and the amino acids required for binding of copper (His-47, -49, -64, and -121) are shaded and boxed orange and zinc (His-64, -72, and -82 and Asp-84) are shaded and boxed purple. The protein kinase C phosphorylation site is boxed blue. Two cysteines (Cys-58 and Cys-147) predicted to be engaged in disulfide bond formation are highlighted red.

```
ATGGTGCTGAAGGCTGTTTGCGTGCTGACAGGAACCGGTGATGTTACCGGGACCGTGTTCTTTGAGCAGGAGgta
 M  V  L  K  A  V  C  V  L  T  G  T  G  D  V  T  G  T  V  F  F  E  Q  E
--------------------------------3496 bp------------------------------------
cctctctgtcatcagGGTGAAGATGCTCCAGTGAAGCTGACCGGGAAGATTGCAGGTCTGGCCCCGGGGGAGCAT
                G  E  D  A  P  V  K  L  T  G  K  I  A  G  L  A  P  G  E  H
GGCTTCCACGTCCACGCCTTCGGAGACAACACCAACGGCTGCATGAGTGCGGGACCCCACTTCAACCCCCACAAC
 G  F  H  V  H  A  F  G  D  N  T  N  G  C  M  S  A  G  P  H  F  N  P  H  N
AAGACCCACGGAGGACCCAACGATGATGTCAGgtgagtctggccttatcactcgtggagttcaggggatagcttt
 K  T  H  G  G  P  N  D  D  V  R
---------------------------------22406 bp-----------------------------------
tgttgctagGCACATAGGGGACCTTGGCAACGTGACTGCAGGAGCTGACAATGTGGCTAAGATCAACATCCAGGA
           H  I  G  D  L  G  N  V  T  A  G  A  D  N  V  A  K  I  N  I  Q  D
CAAGATACTGACTCTCACTGGACCCCTCTCGATCATTGGCAGGACCATGGTGgtaagaacacattgcctgacctg
  K  I  L  T  L  T  G  P  L  S  I  I  G  R  T  M  V
----------------------------------688 bp-----------------------------------
ctcgctctacaccaatagATCCATGAGAAAGCTGATGACCTGGGGAAAGGAGGCAATGAGGAGAGTCTGAAGACG
                   I  H  E  K  A  D  D  L  G  K  G  G  N  E  E  S  L  K  T
GGCAACGCTGGCGGTCGCCAGGCCTGTGGTGTTATTGGAATTACCCAGTAA
 G  N  A  G  G  R  Q  A  C  G  V  I  G  I  T  Q  -
```

Figure S15: Compiled full-length Chinook salmon SOD1b genomic sequence taken from LG30 (GenBank accession No. NC_056458.1) with the cDNA sequence for SOD1b (GenBank accession no. OP760295) highlighted and predicted amino acid sequences shown. The open reading frame is shown in upper-case letters and introns are in lowercase. Within the amino acid sequence, the two Cu/Zn SOD family signatures are underlined (_) and the amino acids required for binding of copper (His-47, -49, -64, and -121) are shaded and boxed orange and zinc (His-64, -72, and -81 and Asp-84) are shaded and boxed purple. The protein kinase C phosphorylation site is boxed blue. Two cysteines (Cys-58 and Cys-147) predicted to be engaged in disulfide bond formation are highlighted red.

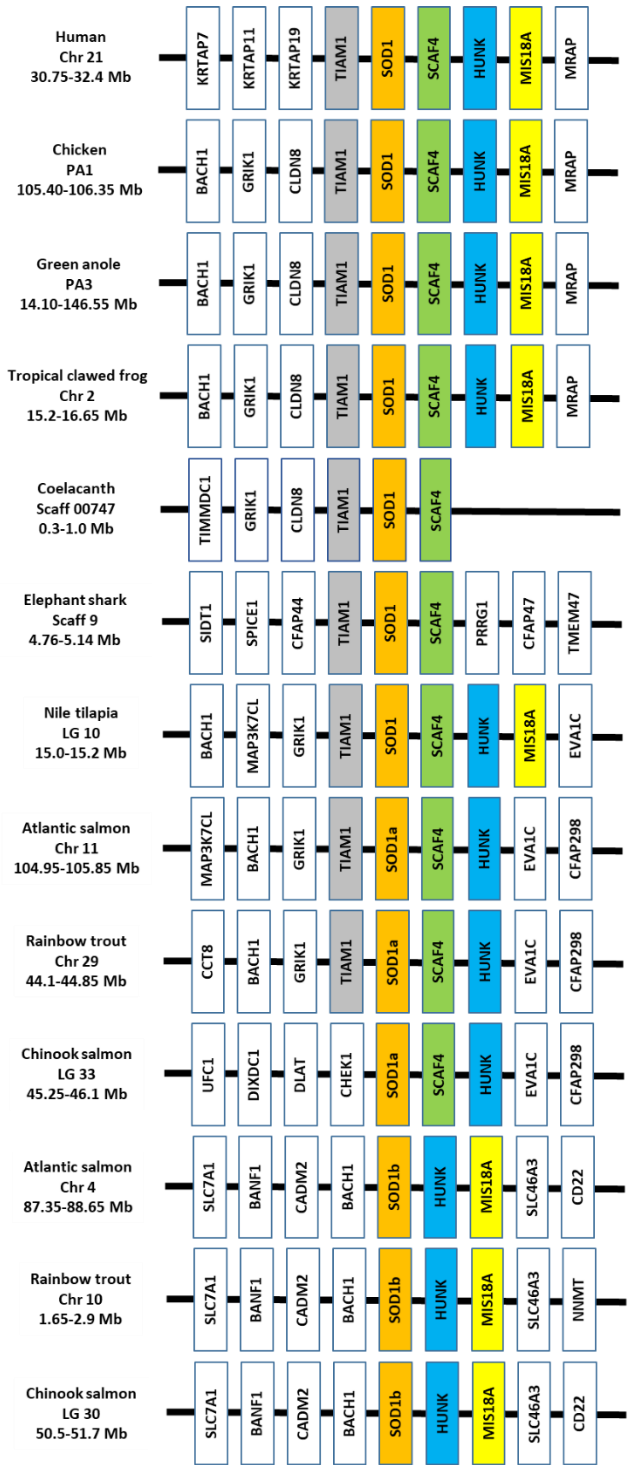

Figure S16: Synteny analysis of the locus containing the SOD1 genes from human, reptile, bird, amphibian and a selection of fish. Two salmonid SOD1 genes (SOD1a and SOD1b) were found split between two different chromosomes. GenBank accession numbers of the fish genomes analysed: Elephant shark Scaffold 118, KI635972.1; Coelacanth Scaffold 00747, JH127308.1; Nile tilapia LG10, NC_031975.2; Atlantic salmon Chromosome 11, NC_059452.1; Rainbow trout Chromosome 29, CM046598.1; Chinook salmon LG33, NC_056461.1; Atlantic salmon Chromosome 4, NC_059445.1; Rainbow trout Chromosome 10, CM046579.1; Chinook salmon LG30, NC_056458.1. The Atlantic salmon, Chinook salmon and rainbow trout SOD1 genes showed conserved linkage with four human chromosome 21 gene: TIAM Rac1 associated GEF 1 (TIAM1), SR-Related CTD Associated Factor 4 (SCAF4), Hormonally up-regulated neu-associated Kinase (HUNK) and MIS18 Kinetochore Protein A (MIS18A).

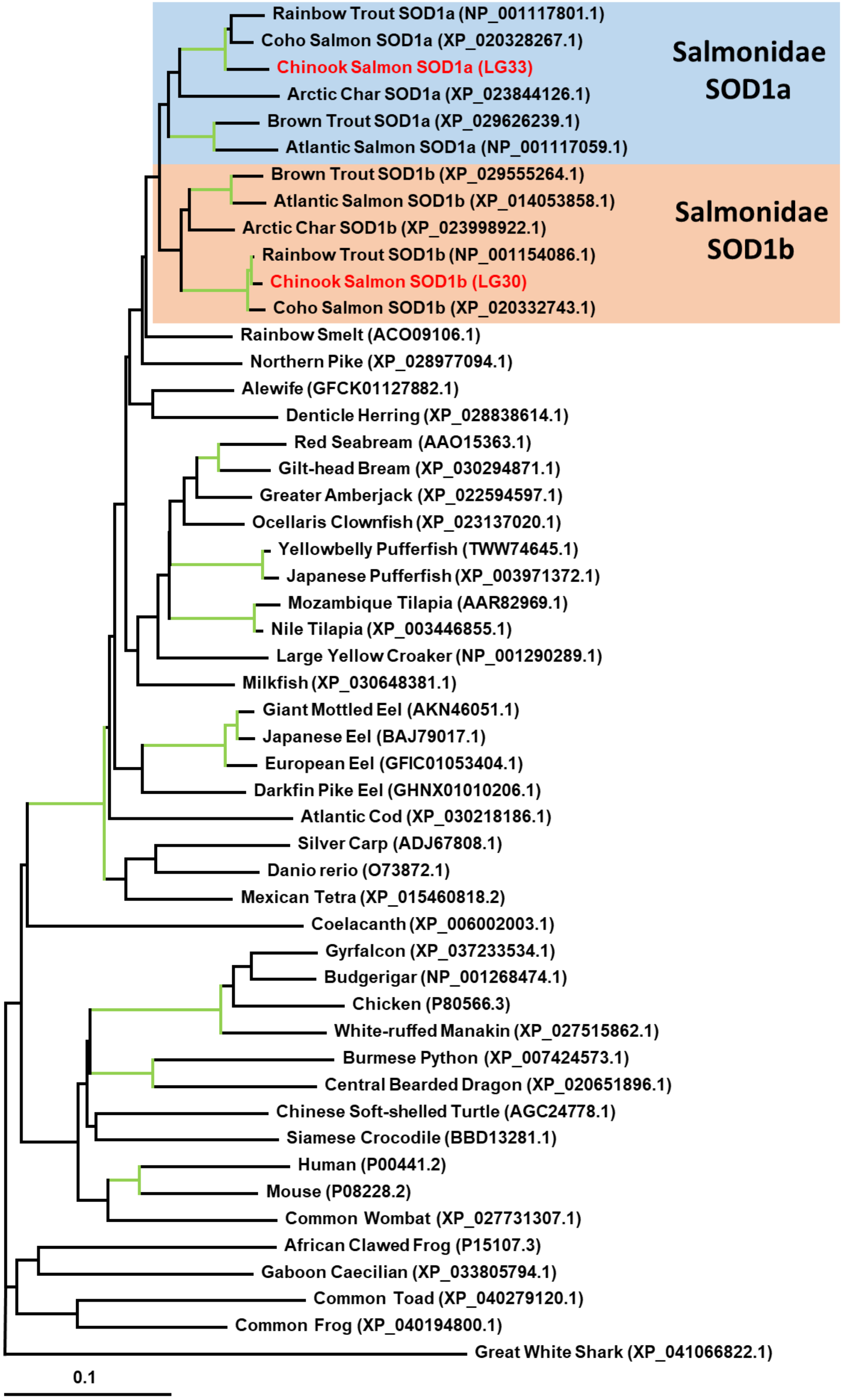

Figure S17: Unrooted phylogenetic tree showing the relationship between the Chinook salmon SOD1a and SOD1b amino acid sequences for the full-length molecule with other known selected vertebrate SOD1 sequences. This tree was constructed by the 'neighbour-joining' method using the Clustal omega and iTOL v6 packages. The tree was bootstrapped 10,000 times and branches supported >75% are highlighted green. The GenBank accession numbers for each organism are shown in brackets. A different colour is used to indicate the clear clustering of the salmonid SOD1 sequences into two distinct groups, SOD1a (Orange) and SOD1b (blue).

```
SOD1a      ATGGCAATGAAGGCTGTTTGCGTGCTCAAAGGCACCGGTGAAGTTACCGGGACCGTATTC    60
SOD1b      ATGGTGCTGAAGGCTGTTTGCGTGCTGACAGGAACCGGTGATGTTACCGGGACCGTGTTC    60
           ****   ******************* * *** ******** ************** ***

SOD1a      TTTGAGCAGGAGGGTGTCGATGGTCCAGTGAAGCTGATTGGGGTGATCTCTGGTCTGGCC    120
SOD1b      TTTGAGCAGGAGGGTGAAGATGCTCCAGTGAAGCTGACCGGGAAGATTGCAGGTCTGGCC    120
           ****************  **** **************  ***  ***  * *********

SOD1a      CCGGGGAAACATGGCTTCCACGTCCATGCTTTTGGAGACAACACCAACGGCTGTATGAGT    180
SOD1b      CCGGGGGAGCATGGCTTCCACGTCCACGCCTTCGGAGACAACACCAACGGCTGCATGAGT    180
           ****** * ***************** ** ** ******************** ******

SOD1a      GCCGGACCCCACTTTAACCCCCACAACAAGACCCACGGAGGACCCACTGATGCTGTTCGG    240
SOD1b      GCGGGACCCCACTTCAACCCCCACAACAAGACCCACGGAGGACCCAACGATGATGTCAGG    240
           ** *********** *******************************  **** ***  **

CSOD1a      CACGTAGGGGACCTTGGCAACGTGACTGCAGGAGCTGACAATGTGGCTAAGATCAACATC   300
CSOD1b      CACATAGGGGACCTTGGCAACGTGACTGCAGGAGCTGACAATGTGGCTAAGATCAACATC   300
            *** ********************************************************

SOD1a      CAGGATGAGATGTTGACTCTCACTGGACCCAACTCTATCATCGGCAGGACCATGGTGATC    360
SOD1b      CAGGACAAGATACTGACTCTCACTGGACCCCTCTCGATCATTGGCAGGACCATGGTGATC    360
           *****  ****  *****************  *** ***** ******************

SOD1a      CATGAGAAGGCTGATGATCTGGAAAAGGAGGCAACGAGGAGAGTCTGAAGACTGGCAAC    420
SOD1b      CATGAGAAAGCTGATGACCTGGGGAAAGGAGGCAATGAGGAGAGTCTGAAGACGGGCAAC    420
           ******** ******** ***** *********** ***************** ******

SOD1a      GCTGGCAGTCGGCAGGCCTGTGGCGTTATTGGCATTGCCCAGTAA    465
SOD1b      GCTGGCGGTCGCCAGGCCTGTGGTGTTATTGGAATTACCCAGTAA    465
           ****** **** *********** ******** *** ********
```

Figure S18: Nucleotide alignment of Chinook salmon SOD1a (GenBank accession no. OP760294) and SOD1b (GenBank accession no. OP760295) cDNA sequences. Exons are highlighted with a different colour. Identical (*) nucleotides identified by the Clustal omega program are indicated. Positions where the forward and reverse primers are found are boxed.

ATGCCGGAAGCCCATGACGCCCCGATGGAGGAAGAGGCAGAGACCTTTGCCTTCCAGGCTGAGATCGCCCAGCTGATGTCCCTGATCATCAACACTTTCT

M P E A H D A P M E E E A E T F A F Q A E I A Q L M S L I I N T F

ACTCCAACAAAGAGATTTTCCTTAGGGAGCTCATCTCCAACTCCTCAGATgtgagtacttcaggcaccacaggcgtttagactacttggtagtttagtat

Y S N K E I F L R E L I S N S S D

--225 bp--

tcaactagGCTCTAGACAAAATCCGCTATGAAAGCCTCACAGACCCATCCAAGATGGACTCTGGCAAGGACCTGAAGATCGAGGTCATTCCCAACAAGGA

A L D K I R Y E S L T D P S K M D S G K D L K I E V I P N K E

GGAGCGCACCCTGACCCTGGTTGACACCGGCATCGGCATGACCAAGGCTGACCTGATCAACAACCTGGGAACCATTGCAAAGTCTGGCACCAAGGCCTTC

E R T L T L V D T G I G M T K A D L I N N L G T I A K S G T K A F

ATGGAGGCCCTGCAGGCTGGGGCTGACATCTCCATGATTGGGCAGTTCGGTGTGGGTTTCTACTCTGCCTACCTGGTGGCTGAGAGGGTGACCGTCATCA

M E A L Q A G A D I S M I G Q F G V G F Y S A Y L V A E R V T V I

CCAAGCACAACGATGATGAGCAGTACATCTGGGAATCATCCGCTGGCGGATCCTTCACCGTCAAAGTCGACCCGTCAGgtaaggacagattatagtttta

T K H N D D E Q Y I W E S S A G G S F T V K V D P S

--135 bp--

tactatagCTGAGTCTATTGGTCGTGGGACCAAGGTGATTCTGTACCTGAAGGATGACCAGACAGAATACTGTGAGGAGAAACGTGTCAAAGAGATCGTG

A E S I G R G T K V I L Y L K D D Q T E Y C E E K R V K E I V

AAGAAGCACTCCCAGTTCATCGGATACCCCATCACACTCTTTgtaagagctagcaggcatgagatagtggaaactccactgagtgattaccaaattgttg

K K H S Q F I G Y P I T L F

--247 bp--

ctgttcagGTGGAGAAGGAGCGTGACAAGGAAGTGAGTGACGATGAGGCAGAGGAGGAAGAGGAGAAGAAGGATGGGGAAGAAGCAGAGAAGAGAGAGGT

V E K E R D K E V S D D E A E E E E E K K D G E E A E K R E V

TGACAAACCCGAGATCGAGGACGTAGGCTCAGACGAGGAGGATCACCATGACCATGACCACGACAGTGCATGCGGTGACAAGAAGAAGAAGAAGAAGAAG

D K P E I E D V G S D E E D H H D H D H D S A C G D K K K K K K K

ATCAAGGAGAAATACATTGACCAGGAGGAGCTGAACAAGACCAAGCCCCTGTGGACCCGTAACCCCGATGACATCACCAACGAGGAGTATGGAGAGTTCT

I K E K Y I D Q E E L N K T K P L W T R N P D D I T N E E Y G E F

ACAAGAGTCTGACCAACGACTGGGAGGAACACCTGGCTGTCAAGgtgaggatattctatttatattggccaacagaataagttcccacgctgtttattta

Y K S L T N D W E E H L A V K

--133 bp--

cttacacagCACTTCTCAGTTGAGGGCCAGCTGGAGTTCCGTGCCCTGCTCTTTGTGCCTCGCCGTGCACCCTTTGACCTCTTTGAAAACAAGAAGAAGA

H F S V E G Q L E F R A L L F V P R R A P F D L F E N K K K

AGAACAATATCAAGCTGTACGTCAGGAGGGTCTTCATCATGGACAACTGTGATGATCTTATCCCTGAGTACCTCAgtgagtcagacagaactgaagcaga

K N N I K L Y V R R V F I M D N C D D L I P E Y L

--72 bp--

tccagACTTCATCAAGGGTGTGGTGGACTCTGAGGATCTCCCCCTAAACATCTCCAGAGAGATGCTGCAGCAGAGCAAGATCCTCAAGGTGATCCGCAAG

N F I K G V V D S E D L P L N I S R E M L Q Q S K I L K V I R K

AACCTGGTCAAGAAGTGTATAGAGCTCTTCACTGAGCTGTCAGAGGACAAAGAAAACTACAAGAAGTACTACGAGCAGTTCTCCAAGAACATCAAGgtca

N L V K K C I E L F T E L S E D K E N Y K K Y Y E Q F S K N I K

--3628 bp--

ttttctctctcccagCTGGGGATCCATGAGGACTCTCAGAACCGTAAGAGGCTGTCAGACATGCTGCGCTACTACTCCTCAGCCTCAGGGGACGAGATGG

L G I H E D S Q N R K R L S D M L R Y Y S S A S G D E M

TATCCCTCAAAGACTATGTCACACGCATGAAGGAAACCCAGAAACATATCTACTACATCACTGgtgaggaaacacacttctctctgtacagcccagttca

V S L K D Y V T R M K E T Q K H I Y Y I T

--51 bp--

atgtttcagGCGAGACCAGAGACCAGGTGGCTAACTCTGCATTTGTGGAACGCCTTCGAAAGGCCGGCCTGGAAGTAATCTACATGATTGAGCCTATTGA

G E T R D Q V A N S A F V E R L R K A G L E V I Y M I E P I D

TGAGTACTGTGTCCAGCAGCTGAAGGAGTATGATGGCAAGACCCTTGTCTCTGTGACCAAGGAGGGTCTGGAGCTGCCTGAGGACGAGGACATGAAGAAG

E Y C V Q Q L K E Y D G K T L V S V T K E G L E L P E D E D M K K

AGGCATGAAGAACAGAAGTCTCAGTTTGAGAACCTCTGCAAGATCATGAAGGACATCTTGGAGAAGAAAGTGGAGAAGgtaagaacgcttaatcacattg

R H E E Q K S Q F E N L C K I M K D I L E K K V E K

--189 bp--

cctttatagGTGACAGTGTCCAACCGCCTGGTCTCCTCCCCCTGCTGCATTGTGACCAGCACCTATGGCTGGACGGCCAACATGGAGAGGATCATGAAGG

V T V S N R L V S S P C C I V T S T Y G W T A N M E R I M K

CCCAGGCCCTGAGGGACAACTCCACCATGGGCTACATGGCTGCCAAGAAGCACCTGGAGATCAACCCAGACCACCCCATTGTGGAGACCCTGAGGCAGAA

A Q A L R D N S T M G Y M A A K K H L E I N P D H P I V E T L R Q K

GGCAGAGGCTGATAAGAATGATAAATCAGTGAAGGACCTAGTCCTTCTGCTGTTTGAGACGGCTCTGTTGTCCTCTGGGTTCACCTTGGATGACCCTCAG

A E A D K N D K S V K D L V L L L F E T A L L S S G F T L D D P Q

ACACACTCCAACCGCATCTACAGAATGATCAAGCTAGGACTGGgtgagtttgtctaaacaagaaccatgtttgctgtcaatatatgatcaatgctagccc

T H S N R I Y R M I K L G L

---162 bp---

cctgtccagGTATTGATGAGGATGACCTGACCCCCGAGGAGCCAACCTCAGCCCCTGTGGAGGACATGCCTCCCCTGGAGGGAGACGAGGACACATCCAG

G I D E D D L T P E E P T S A P V E D M P P L E G D E D T S R

GATGGAGGAGGTTGACTAG

M E E V D -

Figure S19: Compiled full-length Chinook salmon HSP90AA1.1a genomic sequence taken from LG05 (GenBank accession no. NC_056433.1) with the cDNA sequence (GenBank accession no. OP760297) highlighted and predicted amino acid sequence shown. The open reading frame is shown in upper-case letters and introns are in lowercase. The ATP binding domain is underlined. The heat shock hsp90 proteins family signature is highlighted red, the conserved “GxxGxG” motif is boxed red and the MEEVD consensus sequence is highlighted grey and boxed blue.

ATGCCAGAGAAAGCCGGCCACACCATGGATGAGGAAGTGGAGACCTTTGCCTTCCAGGCTGAGATCGCCCAGCTGATGTCTCTGATCATCAACACCTTCT

M P E K A G H T M D E E V E T F A F Q A E I A Q L M S L I I N T F

ATTCCAACAAAGAGATCTTCCTTAGGGAGCTCATCTCCAACTCTTCAGATgtgagtagattaatcataatgctgaattgaccaatgtaacattttaccac

Y S N K E I F L R E L I S N S S D

---142 bp---

gccatgatctgacagGCTTTGGACAAGATCAGATACGAGAGCTTGACAGACCCAACCAAATTGGATTCTTGCAAGGAGCTGAAGATCGAGGTCACCCCTG

A L D K I R Y E S L T D P T K L D S C K E L K I E V T P

ACCTGCGCACTCGTACCCTGACCCTGGTTGACACCGGCATCGGCATGACCAAGGCCGACCTGATCAACAACCTGGGAACCATTGCAAAGTCTGGCACCAA

D L R T R T L T L V D T G I G M T K A D L I N N L G T I A K S G T K

GGCCTTCATGGAGGCCCTGCAGGCTGGAGCTGACATCTCCATGATCGGGCAGTTCGGTGTGGGTTTCTACTCTGCCTACCTGGTGGCTGAGAGGGTGACT

A F M E A L Q A G A D I S M I *G Q F G V G* F Y S A Y L V A E R V T

GTCATCACCAAGCACAATGATGATGAGCAGTACATCTGGGAGTCTGCAGCTGGTGGCTCTTTCACTGTCAAAGTTGACACTGgtaagcatcttcaacagt

V I T K H N D D E Q Y I W E S A A G G S F T V K V D T

---126 bp---

gtctgatttttctgatctgaagGTGAGTCCATTGGCCGTGGCACCAGAGTAATACTGCACATGAAGGAGGACCAGTTTGAATACTGTGAGGAGAAGCGCG

G E S I G R G T R V I L H M K E D Q F E Y C E E K R

TCAAGGAGGTTGTGAAGAAGCACTCTCAGTTCATTGGCTATCCCATCACACTCTTTgtaagttcaaatagaactttatatttctttggttgtaacaatgt

V K E V V K K H S Q F I G Y P I T L F

---123 bp---

cattttcaaccagGTGGAGAAGTCTAGAGAGAAAGAGGTGGACCTTGAGGAGGGAGAAAAGGATGAGGAGGCTGATAAAGATTCTGCAGCTGAGGACCAA

V E K S R E K E V D L E E G E K D E E A D K D S A A E D Q

GATAAGCCCAAGATCGAAGATGTCGGTTCTGATGAGGATGAGGACACCAAGGATTCCAAGAACAAGAGGAAGAAGAAGGTCAAGGAGAAGTACATTGATG

D K P K I E D V G S D E D E D T K D S K N K R K K K V K E K Y I D

CAGAAGAGCTGAATAAGACCAAGCCTATCTGGACCCGTAACCCTGATGACATCACCAATGAGGAGTACGGAGAGTTCTACAAGAGTCTGACCAACGACTG

A E E L N K T K P I W T R N P D D I T N E E Y G E F Y K S L T N D W

GGAGGACCACCTGGCTATCAAGgtgagtctctatgatggcaaactgaaaatcaaaactcaaaccttatgggtttgtcttgatccaacctatgaattaata

E D H L A I K

---34 bp---

attattcctacagCATTTCTCAGTGGAGGGCCAGCTGGAGTTCCGCGCTCTGCTTTTTGTGCCAAGGAGGGCTTCCTTCGACCTCTTCGAGAACAAGAAG

H F S V E G Q L E F R A L L F V P R R A S F D L F E N K K

AAGAAGAACAACATCAAGCTGTACGTGCGCAGGGTGTTCATCATGGACAACTGTGACGAGCTGATGCCAGAGTATCTCAgtgagtactgtaccttgaacc

K K N N I K L Y V R R V F I M D N C D E L M P E Y L

---96 bp---

tccagACTTCATTAAGGGTGTGGTGGACTCTGAGGATCTCCCCCTGAACATCTCCAGAGAGATGCTGCAGCAGAGCAAGATCCTCAAGGTGATCCGCAAG

N F I K G V V D S E D L P L N I S R E M L Q Q S K I L K V I R K

AACCTGGTCAAAAAGTGTATGGATCTTTTCGTCGAGCTCTCAGAAGACAAGGACAACTACAAGAAGTTCTATGAGCAGTTCTCCAAGAACATCAAGgtaa

N L V K K C M D L F V E L S E D K D N Y K K F Y E Q F S K N I K

---98 bp---

catctttccattagCTGGGAATCCATGAAGATGCTCAGAACCGCAAGAAGCTGTCAGACATGCTGCGCTACTACACCTCCAACTCCAACGCTGACGAAAT

L G I H E D A Q N R K K L S D M L R Y Y T S N S N A D E M

GGTCTCCCTGAAGGAGTATGTTTCTCGCATGAAGGACACCCAGAAACACATCTACTACATAACTGgtgagctgctatctattttcactctccattattcc

V S L K E Y V S R M K D T Q K H I Y Y I T

---73 bp---

ttctcttctagGTGAGACCAAGGAACAGGTCGCCAACTCTTCCTTTGTAGAGCGCCTCCGCAAGGCCGGCTTGGAAGTAATCTACATGATTGAACCCATT

G E T K E Q V A N S S F V E R L R K A G L E V I Y M I E P I

GATGAGTACTGTGTCCAGCAGCTGAAGGAGTATGATGGCAAGAACCTGGTCTCTGTGACCAAGGAGGGTCTGGAGCTGCCTGAGGATGAGGATGAGAAGA

D E Y C V Q Q L K E Y D G K N L V S V T K E G L E L P E D E D E K

AGAAGCAGGAGGAGCTGAACACTAAATTCGAGAACCTCTGCAAGACCATGAAGGACATCCTGGACAAGAAGATTGAGAAGgtacaagcacaactcacctg

K K Q E E L N T K F E N L C K T M K D I L D K K I E K

---64 bp---

```
atatgtctacagGTTTCAGTTTCCAACCGCCTGGTCTCCTCCCCCTGTTGCATCGTCACCAGTACATACGGCTGGACGGCCAACATGGAGAGAATCATGA
            V  S  V  S  N  R  L  V  S  S  P  C  C  I  V  T  S  T  Y  G  W  T  A  N  M  E  R  I  M
AATCTCAAGCTCTCAGAGACAACTCCACCATGGGCTACATGACAGCCAAGAAGCACCTGGAGATCAACCCAACCCACCCTATTGTCGAGACTTTGAGAGA
K  S  Q  A  L  R  D  N  S  T  M  G  Y  M  T  A  K  K  H  L  E  I  N  P  T  H  P  I  V  E  T  L  R  E
GAAAGCTGAAGCTGACAAGAACGACAAAGCCGTAAAGGACTTGGTCATCTTGCTGTTCGAGACTGCTCTATTGTCATCTGGGTTCACGCTGGACGACCCT
  K  A  E  A  D  K  N  D  K  A  V  K  D  L  V  I  L  L  F  E  T  A  L  L  S  S  G  F  T  L  D  D  P
CAGACCCATGCAAACCGCATTTACAGGATGATTAAGCTTGGCCTGGgtgagttgctttgccttaatgcattatttgccctcaacttttgtcttggttgat
 Q  T  H  A  N  R  I  Y  R  M  I  K  L  G  L
--------------------------------------------------170 bp----------------------------------------------
ttgcctctacagGCATCGATGGTGATGACTCAGCTGTGGAGGAAATCCTCCAGCCCAGTGAGGATGACATGCCTGTCTTGGAGGGAGATGATGACACATC
            G  I  D  G  D  D  S  A  V  E  E  I  L  Q  P  S  E  D  D  M  P  V  L  E  G  D  D  D  T  S
AAGAATGGAGGAAGTTGACTAA
  R  M  E  E  V  D  -
```

Figure S20: Compiled full-length Chinook salmon HSP90AA1.2a genomic sequence taken from LG05 (GenBank accession no. NC_056433.1) with the cDNA sequence (GenBank accession no. OP760296) highlighted and predicted amino acid sequence shown. The open reading frame is shown in upper-case letters and introns are in lowercase. The ATP binding domain is underlined. The heat shock hsp90 proteins family signature is highlighted red, the conserved “GxxGxG” motif is boxed red and the MEEVD consensus sequence is highlighted grey and boxed blue.

ATGCCGGAAGCCCATGACGCTCCGATGGAGGAGGAGGCAGAGACCTTTGCCTTCCAGGCTGAGATCGCCCAGTTGATGTCGCTGATCATCAACACATTCT

M P E A H D A P M E E E A E T F A F Q A E I A Q L M S L I I N T F

ACTCCAACAAAGAGATCTTCCTTAGGGAGCTCATCTCCAACTCCTCAGATgtgagtacttcagacaccacaagcacttagacttcttggtagtttagtat

Y S N K E I F L R E L I S N S S D

---232 bp---

aatcgactactagGCTCTGGACAAAATCCGCTATGAGAGCCTGACAGACCCGACCAAGATGGACTCTGGCAAGGACCTGAAGATCGAGGTCATTCCCAAC

A L D K I R Y E S L T D P T K M D S G K D L K I E V I P N

AAGGAGGAGCGCACCCTGACCCTGGTTGACACCGGCATCGGCATGACCAAGGCCGACCTGATAAACAACCTGGGAACCATCGCCAAGTCTGGCACCAAGG

K E E R T L T L V D T G I G M T K A D L I N N L G T I A K S G T K

CCTTCATGGAGGCCCTGCAGGCTGGAGCTGACATCTCTATGATCGGGCAGTTCGGTGTGGGTTTCTACTCCGCCTACCTGGTGGCTGAGAGGGTGACTGT

A F M E A L Q A G A D I S M I G Q F G V G F Y S A Y L V A E R V T V

CATCACCAAGCACAATGATGATGAGCAGTACATCTGGGAATCCTCTGCTGGCGGATCCTTCACTGTCAAAGTCGACACGTCAGgtaaagacagattatag

I T K H N D D E Q Y I W E S S A G G S F T V K V D T S

---79 bp---

cccatgtctctctcctatagCTGAGTCTATTGGTCGTGGCACCAAGGTGATTCTGTACCTGAAGGATGACCAGACAGAATATTGTGAGGAGAAACGGGTC

A E S I G R G T K V I L Y L K D D Q T E Y C E E K R V

AAAGAAATCGTGAAGAAGCACTCCCAGTTCATCGGATACCCCATCACACTCTTTgtaagacctagtaggcgtgagtggattaaattgtaactagtgtgaa

K E I V K K H S Q F I G Y P I T L F

---244 bp---

tcctctctgttcagGTGGAGAAGGAGCGTGACAAGGAAGTGAGTGATGATGAGGCGGAGGAGGAGGAGAAGGAGAAGAAAGATGGGGAAGGAGAGGAGGA

V E K E R D K E V S D D E A E E E E K E K K D G E G E E D

CAAACCTGATATTGAGGATGTAGGCTCGGACGAGGAGGATGACCATGATCATGGCCACGACGGCGCATGTGGGGACAAGAAGAAGAAGAAGACGAAGAAG

K P D I E D V G S D E E D D H D H G H D G A C G D K K K K K T K K

ATCAAGGAGAAGTACATTGACCAGGAGGAGCTGAACAAGACCAAGCCCCTTTGGACTCGTAACCCTGATGACATCACCAACGAGGAGTACGGAGAGTTCT

I K E K Y I D Q E E L N K T K P L W T R N P D D I T N E E Y G E F

ACAAGAGCCTGACCAACGACTGGGAGGAACACCTGGCTGTCAAGgtgaggatattctatttatattggccaacataatacatttccacactatttacaaa

Y K S L T N D W E E H L A V K

---125 bp---

tttcttctacagCACTTCTCAGTGGAGGGCCAGCTGGAGTTCCGTGCCCTGCTCTTTGTGCCTCGCCGTGCACCCTTTGACCTCTTTGAGAACAAGAAGA

H F S V E G Q L E F R A L L F V P R R A P F D L F E N K K

AGAAGAACAACATCAAGCTGTACGTCAGGAGGGTCTTCATCATGGACAACTGTGACGATCTGATCCCTGAGTACCTCAgtaagtcagatagaactgaagt

K K N N I K L Y V R R V F I M D N C D D L I P E Y L

---79 bp---

tccagACTTCATCAAGGGTGTGGTGGACTCTGAGGATCTCCCCCTGAACATCTCCAGAGAGATGCTGCAGCAGAGCAAGATCCTCAAGGTGATCCGCAAG

N F I K G V V D S E D L P L N I S R E M L Q Q S K I L K V I R K

AACCTGGTCAAGAAGTGTATAGAGCTTTTCACAGAACTCTCAGAGGACAGAGATAACTACAAGAAGTACTACGAGCAGTTCTCCAAGAACATCAAGgtca

N L V K K C I E L F T E L S E D R D N Y K K Y Y E Q F S K N I K

---768 bp---

ctttcccagCTGGGTATCCATGAGGACTCTCAGAACCGTAAGAGGCTGTCAGACATGCTGCGCTACTACACCTCAGCCTCAGGGGACGAGATGGTCTCCC

L G I H E D S Q N R K R L S D M L R Y Y T S A S G D E M V S

TAAAGGACTACGTCACACGCATGAAGGACACCCAGAAACACATCTACTACATCACTGgtgaggaaacactcctcactgtacagcccagttcataggttgt

L K D Y V T R M K D T Q K H I Y Y I T

---42 bp---

catgttccagGCGAGACCAAAGACCAGGTGGCCAACTCCGCATTCGTGGAGCGCCTTCGCAAGGCTGGCCTGGAGGTGATCTACATGATTGAGCCCATTG

G E T K D Q V A N S A F V E R L R K A G L E V I Y M I E P I

ATGAGTACTGTGTCCAGCAGCTGAAGGAGTACGATGGCAAGAACCTGGTCTCTGTGACCAAGGAGGGTCTGGAGCTGCCTGAGGATGAAGACATGAAGAA

D E Y C V Q Q L K E Y D G K N L V S V T K E G L E L P E D E D M K K

GAGACAAGAAGAGCAGAAGTCTCAGTTTGAGAACCTCTGCAAGATCATGAAGGACATCCTGGAGAAGAAAGTTGAGAAGgtgaggatgcttaatcacact

R Q E E Q K S Q F E N L C K I M K D I L E K K V E K

---176 bp---

acctccccccag**GTGACAGTGTCCAACCGCCTGGTCTCCTCCCCCTGCTGCATCGTGACCAGCACCTACGGCTGGACGGCCAACATGGAGAGGATCATGA**

V T V S N R L V S S P C C I V T S T Y G W T A N M E R I M

AGGCCCAGGCCCTGAGGGACAACTCCACCATGGGCTACATGGCTGCCAAGAAACACCTGGAGATCAACCCAGACCACCCCATTGTGGAGACCCTGAGGCA

K A Q A L R D N S T M G Y M A A K K H L E I N P D H P I V E T L R Q

GAAGGCAGAGGCTGATAAGAATGATAAGTCTGTGAAGGACCTGGTCATTCTGCTGTTTGAGACGGCTCTACTGTCCTCTGGGTTCACCTTGGATGACCCT

K A E A D K N D K S V K D L V I L L F E T A L L S S G F T L D D P

CAGACACACTCCAACCGAATCTACCGGATGATAAAGCTAGGCCTGGgttagttgatttgctcaaacaaggacgatgtttgtgatcaattgtaacccctttt

Q T H S N R I Y R M I K L G L

--524 bp--

cccctcttctcctgtccag**GTATTGACGAGGATGAGCTGACCCCTGAGGAGCCAACTTCAGCCCCCATCGAGGACATGCCTCAACTGGAGGGAGATGAGG**

G I D E D E L T P E E P T S A P I E D M P Q L E G D E

ATACATCCAGGATGGAGGAGGTTGACTAG

D T S R M E E V D -

Figure S21: Compiled full-length Chinook salmon HSP90AA1.1b genomic sequence taken from LG18 (GenBank accession no. NC_056446.1) with the cDNA sequence (GenBank accession no. OP760298) highlighted and predicted amino acid sequence shown. The open reading frame is shown in upper-case letters and introns are in lowercase. The ATP binding domain is underlined. The heat shock hsp90 proteins family signature is highlighted red, the conserved “GxxGxG” motif is boxed red and the MEEVD consensus sequence is highlighted grey and boxed blue.

ATGCCAGAGATAGCCAGCCACACCATGGAGGAGGAAGTGGAGACCTTTGCCTTCCAGGCTGAGATAGCCCAGCTGATGTCCCTGATCATCAACACCTTCT

M P E I A S H T M E E E V E T F A F Q A E I A Q L M S L I I N T F

ACTCCAACAAAGAGATCTTCCTTAGGGAGCTCATCTCCAACTCCTCAGATgtgagtagattaatcataatgctgaattgtcccatgtaacattttacctt

Y S N K E I F L R E L I S N S S D

---130 bp---

acctctttttttcatgttctgacagGCTTTGGACAAGATCAGATATGAGAGCTTGACAGATCCCACCAAATTGGATTCCTGCAAGGACCTAAAGATCGAG

A L D K I R Y E S L T D P T K L D S C K D L K I E

GTCACCCCTGACCTGCGCACTCGCACCCTGACCCTGGTTGACACCGGCATCGGCATGACCAAGGCCGACCTAATAAACAACCTGGGAACCATCGCCAAGT

V T P D L R T R T L T L V D T G I G M T K A D L I N N L G T I A K

CTGGCACCAAGGCCTTCATGGAGGCCCTGCAGGCTGGAGCTGACATCTCTATGATCGGGCAGTTCGGTGTGGGTTTCTACTCCGCCTACCTGGTGGCTGA

S G T K A F M E A L Q A G A D I S M I G Q F G V G F Y S A Y L V A E

GAGGGTGACTGTCATCACCAAGCACAATGATGATGAGCAGTACATCTGGGAGTCTGCAGCTGGTGGCTCCTTCACTGTCAAAGTTGACACTGgtaagcct

R V T V I T K H N D D E Q Y I W E S A A G G S F T V K V D T

---130 bp---

gtgtgagtctgatttgtccgacctgaagGTGAGTCCATTGGCCGTGGCACCAAAGTGATCCTGCACATGAAGGAGGACCAGTTTGAATACTGTGAGGAGA

G E S I G R G T K V I L H M K E D Q F E Y C E E

AGCGCGTCAAGGAGGTTGTGAAGAAGCACTCCCAGTTCATTGGCTACCCCATCACACTCTATgtaagttctaatagagctctatatttctttagtcgtaa

K R V K E V V K K H S Q F I G Y P I T L Y

---100 bp---

cttaaaatgttttaatcacatgaacattttcatcaagGTGGAGAAGTCTAGAGAGAAGGAGGTGGACCTTGAGGAGGGAGAAAAGGATGAGGAGGCTGAT

V E K S R E K E V D L E E G E K D E E A D

AAAGATGCTGCAGCTGAGGACAAAGACAAGCCCAAGATCGAGGACGTCGGCTCTGATGAGGACGAGGACACCAAGGACAGCAAGAACAAGAGGAAGAAGA

K D A A A E D K D K P K I E D V G S D E D E D T K D S K N K R K K

AGGTCAAGGAGAAGTACATCGACGCAGAGGAGCTGAACAAGACCAAGCCTATCTGGACCCGTAACCCTGATGACATCACCAATGAGGAGTACGGAGAGTT

K V K E K Y I D A E E L N K T K P I W T R N P D D I T N E E Y G E F

CTACAAGAGTCTGACCAACGACTGGGAGGACCACCTGGCTATCAAGgtgagtctatgatagcaacctgaaaactcttgatccaacctatgaattaatata

Y K S L T N D W E D H L A I K

--30 bp---

tttttattcccacagCACTTCTCAGTGGAGGGCCAGCTGGAGTTCCGCGCTCTGCTCTTTGTGCCCAGGAGGGCTTCCTTTGACCTCTTTGAGAACAAGA

H F S V E G Q L E F R A L L F V P R R A S F D L F E N K

AGAAGAAGAACAACATCAAGCTGTATGTGCGCAGGGTCTTCATCATGGATAACTGTGACGAGCTGATGCCAGAGTATCTCAgtgagtactgttccttaaa

K K K N N I K L Y V R R V F I M D N C D E L M P E Y L

--94 bp---

ccagACTTCATCAAGGGTGTGGTGGACTCTGAGGATCTCCCCCTGAACATCTCCAGAGAGATGCTGCAGCAGAGCAAGATCCTCAAGGTGATCCGCAAGA

N F I K G V V D S E D L P L N I S R E M L Q Q S K I L K V I R K

ACCTGGTCAAGAAGTGTATAGAGCTTTTCATCGAGCTCTCAGAGGACAAGGACAATTACAAGAAGTTCTACGAGCAGTTCTCTAAGAACATCAAGgtacg

N L V K K C I E L F I E L S E D K D N Y K K F Y E Q F S K N I K

---100 bp---

gttgtgtaccttttagCTGGGAATCCATGAGGACTCTCAGAACCGCAAGAAATTGTCAGACATGCTGCGCTACTACACCTCCAACTCCGGTGATGAAATG

L G I H E D S Q N R K K L S D M L R Y Y T S N S G D E M

GTTTCCTTGAAGGACTACGTTTCCCGCATGAAGGACACCCAGAAACACATCTACTACATTACTGgtgagcttccctacatttttctctccattatttt

V S L K D Y V S R M K D T Q K H I Y Y I T

--65 bp---

ctgtgtatcctcttctagGTGAGACGAAGGAACAGGTCGCCAACTCTTCCTTTGTGGAGCGCCTCCGCAAGGCCGGCCTGGAAGTGATCTACATGATTGA

G E T K E Q V A N S S F V E R L R K A G L E V I Y M I E

GCCCATTGATGAGTACTGTGTCCAGCAGCTGAAGGAGTATGATGGCAAGAACCTAGTCTCTGTGACCAAGGAGGGTCTGGAGCTGCCTGAGGATGAGGAT

P I D E Y C V Q Q L K E Y D G K N L V S V T K E G L E L P E D E D

GAGAAAAAGAAACAGGAGGAGCTGAATTCTAAATTTGAGAACCTTTGCAAGATCATGAAGGACATCCTGGACAAGAAAATTGAGAAGgtacaagcacaat

E K K K Q E E L N S K F E N L C K I M K D I L D K K I E K

---100 bp---

gacag**GTTTCAGTGTCCAACCGCCTGGTCTCCTCCCCCTGCTGCATTGTGACCAGCACCTACGGGTGGACAGCCAACATGGAGAGGATCATGAAATCTCA**

V S V S N R L V S S P C C I V T S T Y G W T A N M E R I M K S Q

AGCTCTCAGAGACAACTCCACCATGGGCTACATGACAGCCAAAAAGCACCTGGAGATCAACCCAACCCACCCTATTGTCGAGACTCTGAGAGAGAAAGCT

A L R D N S T M G Y M T A K K H L E I N P T H P I V E T L R E K A

GAGGCCGACAAGAACGACAAAGCCGTGAAGGACCTGGTCATCTTGCTATTCGAGACTGCTCTGATGTCTTCTGGATTCACACTGGACGACCCTCAGACCC

E A D K N D K A V K D L V I L L F E T A L M S S G F T L D D P Q T

ACGCAAATCGCATCTACAGGATGATCAAGCTTGGCCTGGgtgagttgatcaagcttattcgccctcagcttttgtcttggttgatgggattgaaagtctc

H A N R I Y R M I K L G L

--150 bp--

atgtaagctatttcttcccatctacag**GCATCGATGATGATGATTCAGCAGTGGAGGACATCCTCCAGCCCAGTGAGGATGACATGCCTGTCCTGGAGGG**

G I D D D D S A V E D I L Q P S E D D M P V L E G

AGATGATGACACCTCTAGAATGGAGGAAGTTGACTAA

D D D T S R M E E V D -

Figure S22: Compiled full-length Chinook salmon HSP90AA1.2b genomic sequence taken from LG18 (GenBank accession no. NC_056446.1) with the cDNA sequence (GenBank accession no. OQ215311) highlighted and predicted amino acid sequence shown. The open reading frame is shown in upper-case letters and introns are in lowercase. The ATP binding domain is underlined. The heat shock hsp90 proteins family signature is highlighted red, the conserved “GxxGxG” motif is boxed red and the MEEVD consensus sequence is highlighted grey and boxed blue.

ATGCCTGAAGAAATGCGCCAAGAGGAGGAGGCTGAGACCTTTGCCTTCCAGGCAGAGATCGCTCAGCTCATGTCCCTGATCATCAACACCTTTTATTCCA

M P E E M R Q E E E A E T F A F Q A E I A Q L M S L I I N T F Y S

ACAAGGAAATCTTCCTCAGGGAGTTGATTTCCAATGCATCTGATgtaagtgagaaggttggcgtttggccccatgttgaatgtgtttataaaacacccct

N K E I F L R E L I S N A S D

--44 bp--

aatgtctttcag**GCTTTGGACAAAATCCGATACGAAAGTCTGACGGACCCCACCAAGCTGGACAACGGCAAGGAACTGAAGATTGACGTCATCCCCAACG**

A L D K I R Y E S L T D P T K L D N G K E L K I D V I P N

TGGAGGAGCGCACCCTGACCCTAATCGACACTGGAATTGGCATGACCAAAGCTGACCTCATCAACAACCTGGGAACCATCGCGAAGTCTGGCACCAAGGC

V E E R T L T L I D T G I G M T K A D L I N N L G T I A K S G T K A

CTTCATGGAGGCCCTGCAGGTATGTCAATTGGATTACAGAAATATTTGCATATGCATTCAAATACTATACAGGGAGAGAACATGTTGTGATTATTTCCCT

F M E A L Q

ctttgctag**GCTGGAGCTGACATCTCCATGATTGGGCAGTTTGGTGTGGGTTTCTACTCTGCCTACCTGGTGGCTGAGAGGGTGACTGTCATCACTAAGC**

A G A D I S M I G Q F G V G F Y S A Y L V A E R V T V I T K

ACAACGATGATGAGCAGTACATCTGGGAGTCCTCAGCCGGAGGCTCCTTCACAGTCAAGGTCGACACTGgtatgtgtcccattcaatgaaagctgggttc

H N D D E Q Y I W E S S A G G S F T V K V D T

---133 bp---

catttccag**GGGAGCCCATGTTGCGTGGAACTAAGGTGATTCTGCACATGAAGGAGGACCAGACGGAGTATGTTGAGGAGAAGAGGGTCAAGGAGGTGGT**

G E P M L R G T K V I L H M K E D Q T E Y V E E K R V K E V V

CAAGAAGCACTCTCAGTTCATTGGATATCCCATCACCCTCTTTgtgagtatatgtatggaagtatttgacaagtgcgattagtgacactttctgtaggtg

K K H S Q F I G Y P I T L F

--77 bp--

tctgtgcag**GTTGAGAAGGAGCGTGAAAAGGAGATCAGTGACGATGAGGCTGAGGAGGAGGAGAAGGCAGAGAAGGAGGAGAAAGAGGAGAAGGAGGCAG**

V E K E R E K E I S D D E A E E E E K A E K E E K E E K E A

AGGACAAGCCCAAGATTGAGGATGTGGGCTCTGATGATGAGGAGGACTCCAAAGACAAGGACAAGAAAAAGACCAAGAAGATCAAGGAGAAGTACATTGA

E D K P K I E D V G S D D E E D S K D K D K K K T K K I K E K Y I D

CCAGGAGGAGCTGAACAAGACCAAGCCCATTTGGACCAGAAACCCTGACGACATCACCATGGAGGAGTACGGAGAGTTCTACAAGAGCCTGACCAACGAC

Q E E L N K T K P I W T R N P D D I T M E E Y G E F Y K S L T N D

TGGGAGGAACACCTGGCTGTCAAGgtacaaacattgaaatttagaaattgcgacaaaatcaaaatggtttcctgtggttgcccagatggcctggtcccag

W E E H L A V K

---110 bp---

cttctacag**CACTTTTCAGTGGAGGGCCAGCTGGAGTTCCGTGCTCTCCTCTTTATCCCCCGCCGCGCACCCTTCGACCTCTTTGAGAACAAGAAGAAGA**

H F S V E G Q L E F R A L L F I P R R A P F D L F E N K K K

AGAACAACATCAAGCTGTATGTCAGGAGGGTCTTCATCATGGACAGCTGTGAGGAGCTCATCCCGGAGTACCTGAgtaagtaacccagtcttcatgtctt

K N N I K L Y V R R V F I M D S C E E L I P E Y L

---331 bp---

gcag**ATTTTGTGCGTGGTGTGGTAGACTCTGAGGATCTCCCCCTGAACATCTCCCGAGAGATGCTGCAGCAGAGCAAGATCCTCAAGGTGATCCGCAAGA**

N F V R G V V D S E D L P L N I S R E M L Q Q S K I L K V I R K

ACATCGTCAAGAAGTGTATGGAGCTGTTCGGCGAGCTGGCAGAGGACAGGGAGAACTACAACAAGTTCTATGATGGCTTCTCCAAGAACCTCAAGgtaac

N I V K K C M E L F G E L A E D R E N Y N K F Y D G F S K N L K

---441 bp---

tctctacag**CTGGGCATCCACGAGGACTCCCAGAACCGCAAGAAGCTGTCGGAGCTGCTGCGCTACCACAGCTCTCAGTCGGGAGACGAGCTGACCTCCC**

L G I H E D S Q N R K K L S E L L R Y H S S Q S G D E L T S

TCACGGAGTACCTCACCCGCATGAAGGACAACCAGAAATCCATCTATTACATCACTGgtgggtctagttagccatttatccccaataaagtagagcccca

L T E Y L T R M K D N Q K S I Y Y I T

--45 bp--

tttttatag**GTGAGAGCAAGGACCAGGTGGCCAACTCTGCTTTTGTGGAGCGCGTGCGCAAGCGAGGATTCGAGGTCCTGTACATGACGGAGCCCATTGA**

G E S K D Q V A N S A F V E R V R K R G F E V L Y M T E P I D

CGAGTACTGTGTCCAGCAGCTGAAGGAGTTTGACGGAAAGACCCTGGTCTCCGTGACCAAGGAGGGCCTGGAGCTGCCGGAGGACGAGGAGGAGAAGAAG

E Y C V Q Q L K E F D G K T L V S V T K E G L E L P E D E E E K K

AAGATGGATGAGGACAAGACAAAGTTTGAGAACCTCTGCAAGCTCATGAAGGAGATCCTGGACAAGAAAGTAGAGAAGgtaggtgactggccaaacctat

K M D E D K T K F E N L C K L M K E I L D K K V E K

---508 bp---

ttcccccag**GTGACCGTGTCAAACAGGCTGGTGTCGTCGCCCTGCTGCATTGTGACCAGCACGTATGGCTGGACGGCAAACATGGAGAGGATCATGAAGG**

V T V S N R L V S S P C C I V T S T Y G W T A N M E R I M K

CGCAGGCCCTGAGGGACAACTCCACCATGGGCTACATGATGGCCAAGAAACACCTGGAGATCAACCCAGACCACCCCATCGTGGAGACCCTGCGGCAGAA

A Q A L R D N S T M G Y M M A K K H L E I N P D H P I V E T L R Q K

GGCTGACCTGGACAAGAACGACAAGGCGGTGAAGGACCTGGTAATCCTGCTTTTCGAGACCGCTCTGCTCTCCTCTGGCTTCAGCCTGGATGACCCTCAA

A D L D K N D K A V K D L V I L L F E T A L L S S G F S L D D P Q

ACTCACTCCAACCGTATCTACAGGATGATCAAGCTGGGCCTGGgtgaggatgcactcaactgtgggaggggtgaatgaaggtaatcttgattataatgg

T H S N R I Y R M I K L G L

---25 bp---

cccctccag**GAATCGATGATGACGAAGTGATCCCTGAGGAGCCCACCTCCGCACCTGCCCCAGATGAGATCCCACCTCTAGAGGGAGACGATGATGCATC**

G I D D D E V I P E E P T S A P A P D E I P P L E G D D D A S

ACGCATGGAGGAAGTGGATTAA

R M E E V D -

Figure S23: Compiled full-length Chinook salmon HSP90AB1a genomic sequence taken from LG05 (GenBank accession no. NC_056433.1) with the cDNA sequence (GenBank accession no. OP760299) highlighted and predicted amino acid sequence shown. The open reading frame is shown in upper-case letters and introns are in lowercase. The ATP binding domain is underlined. The heat shock hsp90 proteins family signature is highlighted red, the conserved "GxxGxG" motif is boxed red and the MEEVD consensus sequence is highlighted grey and boxed blue.

ATGCCTGAAGAAATGCGCCAAGAGGAGGAGGCTGAGACCTTTGCCTTCCAGGCAGAGATTGCTCAGCTCATGTCCCTGATCATCAACACCTTTTATTCCA

M P E E M R Q E E E A E T F A F Q A E I A Q L M S L I I N T F Y S

ACAAGGAGATCTTCCTCAGGGAGTTGATCTCCAATGCCTCTGATgtaagttttttttttttttttgttaggggtgtatcatgctgttcaaagtctaattg

N K E I F L R E L I S N A S D

---187 bp---

tttcagGCTTTGGACAAAATCCGCTACGAAAGTCTGACGGACCCCACCAAGCTGGACAATGGCAAGGAATTGAAGATTGACATCATCCCCAATGTGGAGG

A L D K I R Y E S L T D P T K L D N G K E L K I D I I P N V E

AACGCACCCTGACCCTCATCGACACTGGAATTGGCATGACCAAAGCTGACCTCATCAACAACCTGGGAACTATTGCCAAGTCTGGCACCAAGGCCTTCAT

E R T L T L I D T G I G M T K A D L I N N L G T I A K S G T K A F M

GGAGGCCCTGCAAgtatgtccatggcattacagcaatattcaataatatgcctcaaatactattcagttgaacattgagagtgattaatttttttcccat

E A L Q

ctttgctagGCTGGAGCTGACATCTCCATGATTGGGCAGTTCGGTGTGGGATTCTACTCCGCCTACCTGGTGGCAGAAAGAGTGACTGTCATCACTAAGC

A G A D I S M I G Q F G V G F Y S A Y L V A E R V T V I T K

ACAATGATGACGAGCAGTACATCTGGGAGTCTTCAGCCGGAGGCTCATTCACAGTCAAGGTCGACAGTGgtatgtgtcttgcccaatgaaggctatgcgg

H N D D E Q Y I W E S S A G G S F T V K V D S

---720 bp---

aatttccagGGGAGCCCATGTTGCGTGGAACTAAGGTAATTCTGCACATGAAGGAGGACCAGACTGAGTATGTTGAGGAGAAGAGGGTCAAGGAGGTGGT

G E P M L R G T K V I L H M K E D Q T E Y V E E K R V K E V V

CAAGAAGCACTCTCAGTTCATTGGATATCCCATCACACTTTTCgtgagtatagaattatgggagtatttataactagtgtgattagtgacatttatgtag

K K H S Q F I G Y P I T L F

---79 bp---

tgtgtgcagGTTGAAAAGGAGCGTGAAAAGGAGATCAGTGATGATGAGGCTGAGGAGAAGGAGGAAAAGGCGGAGGAAAAGGCGGAGGCTGAGGACAAGC

V E K E R E K E I S D D E A E E K E E K A E E K A E A E D K

CCAAAATTGAGGATGTGGGCTCGGATGACGAGGAGGATTCCAAAGACAAGGACAAGAAGAAGACTAAGAAGATAAAGGAGAAGTACATTGACCAGGAGGA

P K I E D V G S D D E E D S K D K D K K K T K K I K E K Y I D Q E E

GCTGAATAAGACCAAGCCCATCTGGACCAGGAACCCTGATGACATCACCATGGAGGAGTATGGGGAGTTCTACAAGAGCCTGACCAATGACTGGGAGGAC

L N K T K P I W T R N P D D I T M E E Y G E F Y K S L T N D W E D

CACCTTGCAGTCAAGgtactatcactaaaatatacataaattacaatgtcaaaatggtatgcccagaaggtattcctaggtggcttggttccagaaatgt

H L A V K

---447 bp---

cttttgcagCACTTCTCAGTGGAGGGCCAGCTGGAGTTCCGTGCCCTGCTCTTTATCCCCCGCCGCGCACCCTTTGACCTCTTTGAGAACAAGAAGAAGA

H F S V E G Q L E F R A L L F I P R R A P F D L F E N K K K

AGAATAACATCAAGCTGTATGTCAGGAGGGTCTTCATCATGGACAGCTGCGAAGAGCTTATCCCAGAGTACCTGAgtaagtagcctagtctccagatagt

K N N I K L Y V R R V F I M D S C E E L I P E Y L

---249 bp---

gcagACTTTGTGCGTGGTGTGGTGGACTCTGAGGATCTCCCCCTGAACATCTCCCGAGAGATGCTGCAACAGAGCAAGATCCTCAAGGTCATCCGCAAGA

N F V R G V V D S E D L P L N I S R E M L Q Q S K I L K V I R K

ACATCGTCAAGAAGTGTATGGAGCTGTTCGGTGAGCTGGCAGAGGACAAGGAGAACTACAAGAAGTTCTACGATGGCTTCTCCAAGAACCTCAAGgtaac

N I V K K C M E L F G E L A E D K E N Y K K F Y D G F S K N L K

---1720 bp---

tctctgcagCTGGGGATCCACGAGGACTCCCAAAACCGCAAGAAGCTGTCCGAGCTGCTTCGCTACCACAGCTCCCAGTCTGGAGATGAGCTGACCTCCC

L G I H E D S Q N R K K L S E L L R Y H S S Q S G D E L T S

TCACAGAGTACCTCACCCGCATGAAGGACAACCAAAAATCCATCTACTACATAACCGgtgggtctagttacacattcaatcaacataactttgagcccta

L T E Y L T R M K D N Q K S I Y Y I T

---60 bp---

tttcttaagGTGAGAGCAAGGACCAGGTGGCCAACTCAGCCTTTGTAGAACGCGTGCGTAAGCGTGGCTTCGAGGTCCTGTACATGACGGAGCCCATCGA

G E S K D Q V A N S A F V E R V R K R G F E V L Y M T E P I D

TGAATACTGCGTCCAGCAGCTGAAGGAGTTTGACGGTAAAACCCTGGTCTCTGTAACCAAGGAGGGCTTGGAGCTGCCTGAGGATGAGGAGGAGAAGAAG

E Y C V Q Q L K E F D G K T L V S V T K E G L E L P E D E E E K K

AAGATGGAGGAGGACAAGACGAGGTTTGAGAACCTCTGCAAGCTCATGAAGGAGATCCTGGACAAGAAAGTAGAGAAGgtaaatgacgggaaccaacctg

K M E E D K T R F E N L C K L M K E I L D K K V E K

---578 bp---

ccccttcag**GTGACTGTGTCAAACAGGCTGGTGTCGTCGCCCTGCTGCATCGTGACCAGCACGTATGGCTGGACGGCCAACATGGAGCGCATCATGAAGG**

V T V S N R L V S S P C C I V T S T Y G W T A N M E R I M K

CACAGGCCCTGAGGGACAACTCCACAATGGGCTACATGATGGCCAAGAAGCACCTGGAGATTAACCCAGACCACCCCATTGTGGAGACCCTAAGGCAGAA

A Q A L R D N S T M G Y M M A K K H L E I N P D H P I V E T L R Q K

GGCTGACCTGGACAAAAACGACAAGGCGGTGAAGGACTTAGTTATCCTATTATTCGAGACTGCATTGCTCTCTTCGGGCTTCAGCCTGGATGACCCCCAG

A D L D K N D K A V K D L V I L L F E T A L L S S G F S L D D P Q

ACTCACTCCAACCGCATCTACAGGATGATCAAGCTGGGTCTGGgtaaggatgcacactcggaactgttagggggaaggaaacggctctaaagggtaacat

T H S N R I Y R M I K L G L

---50 bp---

ccctttcag**GAATCGATGATGACGAAGTGATCCCCGAGGAACCCACCTCTGCACCCGCCCCAGATGAGATCCCACCTCTAGAGGGAGATGAGGATGCTTC**

G I D D D E V I P E E P T S A P A P D E I P P L E G D E D A S

ACGCATGGAGGAAGTGGATTAA

R M E E V D -

Figure S24: Compiled full-length Chinook salmon HSP90AB1b genomic sequence taken from LG18 (GenBank accession no. NC_037114.1) with the cDNA sequence (GenBank accession no. OP760300) highlighted and predicted amino acid sequence shown. The open reading frame is shown in upper-case letters and introns are in lowercase. The ATP binding domain is underlined. The heat shock hsp90 proteins family signature is highlighted red, the conserved “GxxGxG” motif is boxed red and the MEEVD consensus sequence is highlighted grey and boxed blue.

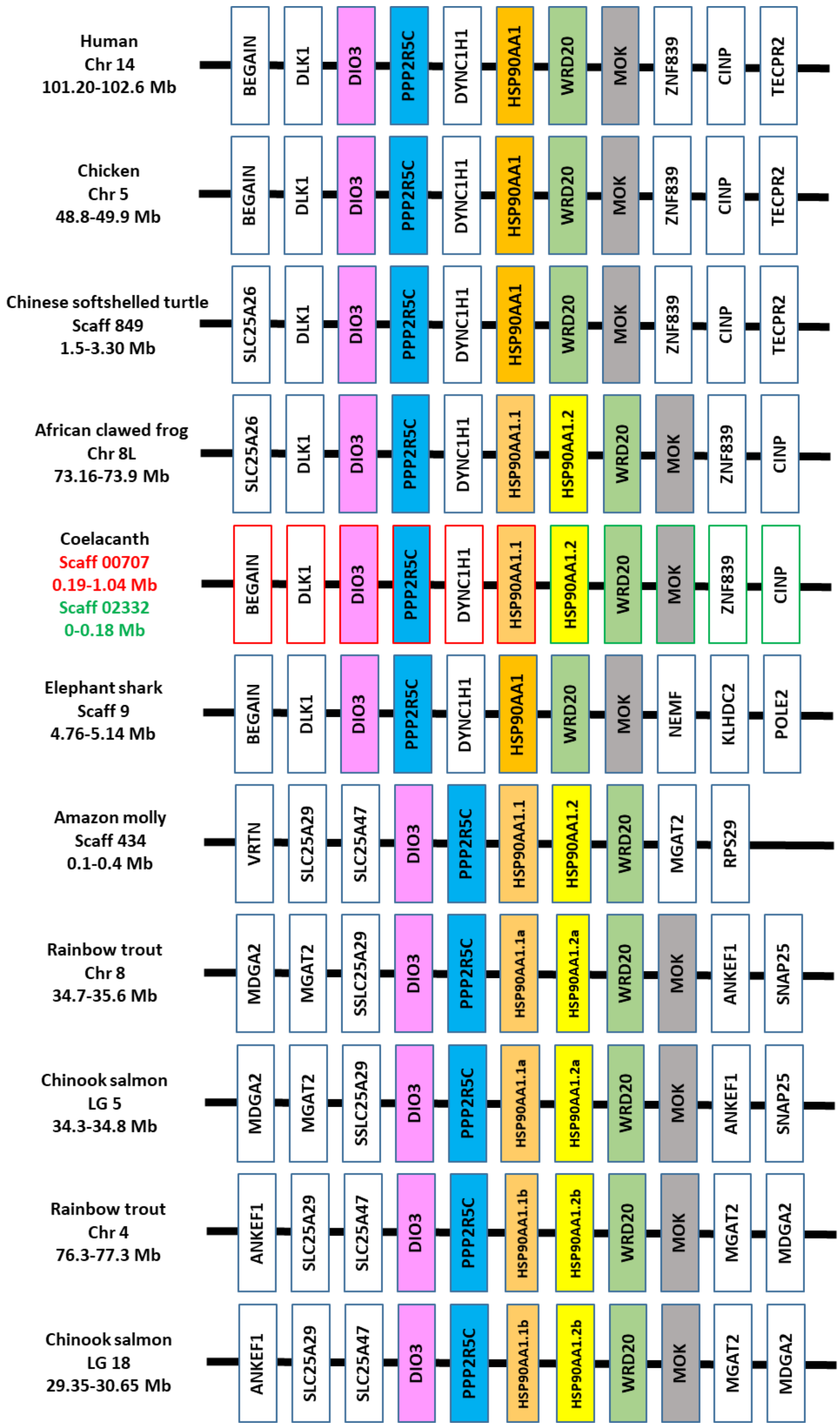

Figure S25: Synteny analysis of the locus containing the HSP90AA1 genes from human, reptile, bird, amphibian and a selection of fish. Four salmonid HSP90AA1 genes (HSP90AA1.1a, HSP90AA1.2a, HSP90AA1.1b and HSP90AA1.2b) were found split between two different chromosomes. GenBank accession numbers of the fish genomes analysed: Elephant shark Scaffold 9, NW_024704750.1; Coelacanth Scaffold 00707, NW_005819717.1; Coelacanth Scaffold 02332, NW_005821342.1; Amazon molly Scaffold 434, NW_006800373.1; Rainbow trout Chromosome 4, CM046573.1; Rainbow trout Chromosome 8, CM046577.1; Chinook Salmon LG05, NC_056433.1; Chinook Salmon LG18, NC_056446.1. Coelacanth synteny was constructed using two separate scaffolds.

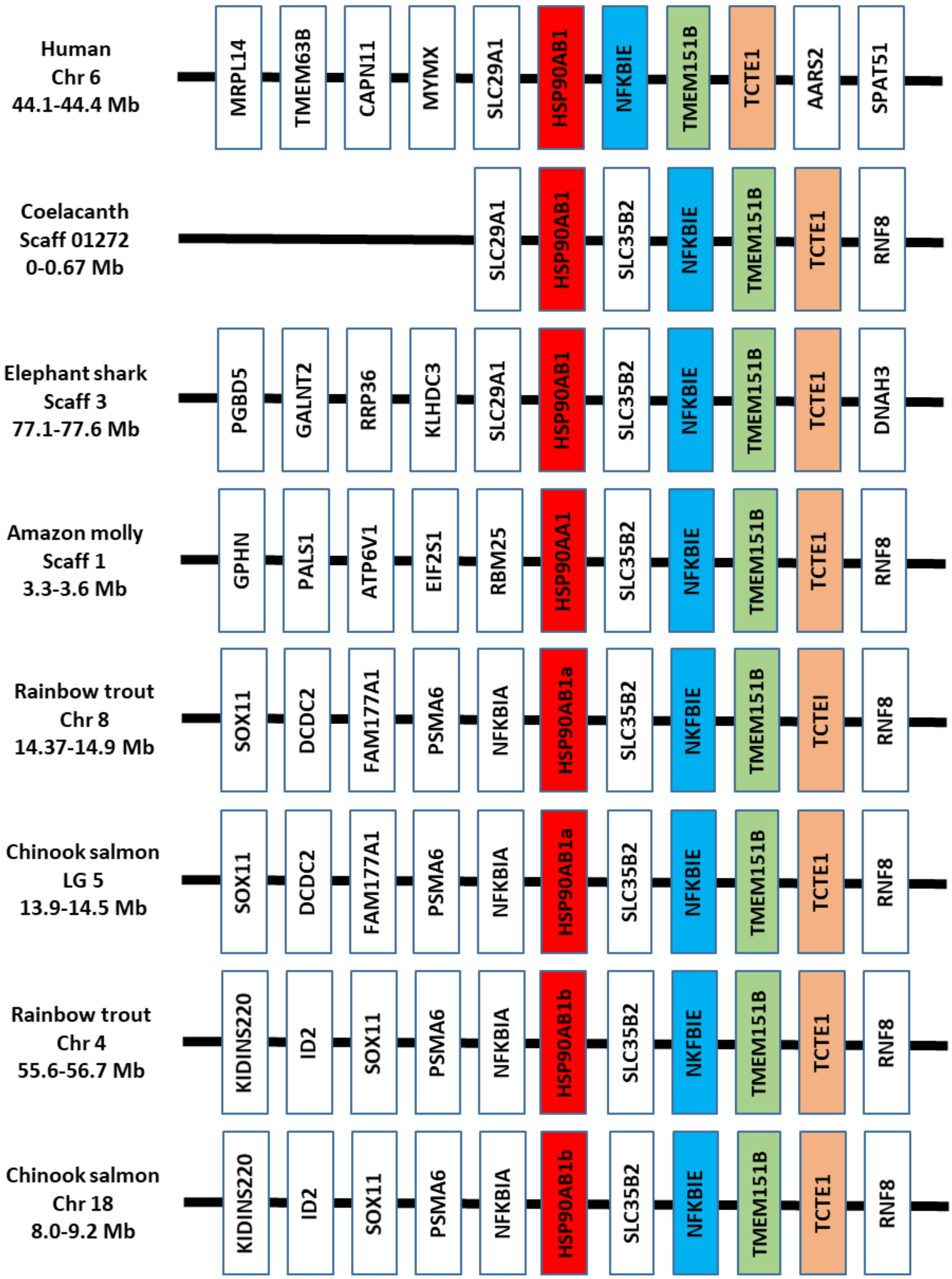

Figure S26. Synteny analysis of the locus containing the HSP90AB1 genes from human and a selection of fish. Two salmonid HSP90AB1 genes (HSP90AB1a and HSP90AB1b) were found split between two different chromosomes. GenBank accession numbers of the fish genomes analysed: Elephant shark Scaffold 3, NW_024704744.1; Coelacanth Scaffold 01272, NW_005820282.1; Amazon molly Scaffold 1, NW_006799940.1; Rainbow trout Chromosome 4, CM046573.1; Rainbow trout Chromosome 8, CM046577.1; Chinook Salmon LG5, NC_056433.1 (Otsh_v2.0); Chinook Salmon LG18, NC_037114.1 (Otsh_v1.0).

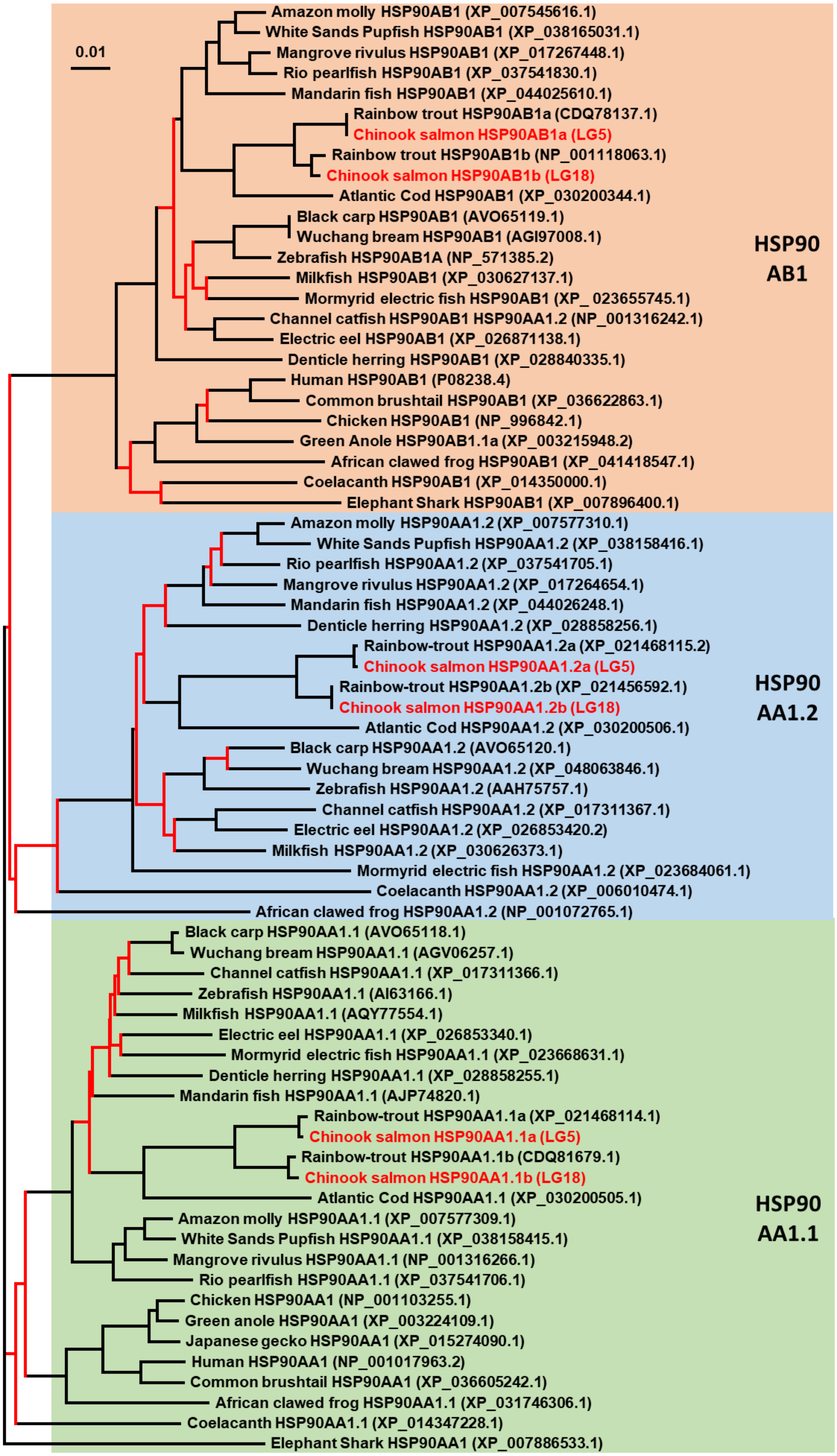

Figure S27: Unrooted phylogenetic tree showing the relationship between the Chinook salmon HSP90AA1 and HSP90AB1 amino acid sequence for the full-length molecule with other known selected vertebrate HSP90AA1 and HSP90AB1 sequences. This tree was constructed by the 'neighbour-joining' method using the Clustal omega and iTOL v6 packages. The tree was bootstrapped 10,000 times and branches supported <75% are highlighted red. The GenBank accession numbers for each organism are shown in brackets.

```
HSP90AA1.1a    ATGCCGGAAGCCCATGACGCCCCGATGGAGGAAGAGGCAGAGACCTTTGCCTTCCAGGCTGAGATCGCCCAGCTGATGTCCCTGATCATC    90
HSP90AA1.2a    ATGCCAGAGAAAGCCGGCCACACCATGGATGAGGAAGTGGAGACCTTTGCCTTCCAGGCTGAGATCGCCCAGCTGATGTCTCTGATCATC    90
HSP90AA1.1b    ATGCCGGAAGCCCATGACGCTCCGATGGAGGAGGAGGCAGAGACCTTTGCCTTCCAGGCTGAGATCGCCCAGTTGATGTCGCTGATCATC    90
HSP90AA1.2b    ATGCCAGAGATAGCCAGCCACACCATGGAGGAGGAAGTGGAGACCTTTGCCTTCCAGGCTGAGATAGCCCAGCTGATGTCCCTGATCATC    90
HSP90AB1a      ATGCCTGAAGAAATG-----CGC-CAAGAGGAGGAGGCTGAGACCTTTGCCTTCCAGGCAGAGATCGCTCAGCTCATGTCCCTGATCATC    84
HSP90AB1b      ATGCCTGAAGAAATG-----CGC-CAAGAGGAGGAGGCTGAGACCTTTGCCTTCCAGGCAGAGATTGCTCAGCTCATGTCCCTGATCATC    84
               ***** **.. .         * .:.** **.**.*  *******************:***** ** *** * ***** *********

HSP90AA1.1a    AACACTTTCTACTCCAACAAAGAGATTTTCCTTAGGGAGCTCATCTCCAACTCCTCAGATGCTCTAGACAAAATCCGCTATGAAAGCCTC    180
HSP90AA1.2a    AACACCTTCTATTCCAACAAAGAGATCTTCCTTAGGGAGCTCATCTCCAACTCTTCAGATGCTTTGGACAAGATCAGATACGAGAGCTTG    180
HSP90AA1.1b    AACACATTCTACTCCAACAAAGAGATCTTCCTTAGGGAGCTCATCTCCAACTCCTCAGATGCTCTGGACAAAATCCGCTATGAGAGCCTG    180
HSP90AA1.2b    AACACCTTCTACTCCAACAAAGAGATCTTCCTTAGGGAGCTCATCTCCAACTCCTCAGATGCTTTGGACAAGATCAGATATGAGAGCTTG    180
HSP90AB1a      AACACCTTTTATTCCAACAAGGAAATCTTCCTCAGGGAGTTGATTTCCAATGCATCTGATGCTTTGGACAAAATCCGATACGAAAGTCTG    174
HSP90AB1b      AACACCTTTTATTCCAACAAGGAGATCTTCCTCAGGGAGTTGATCTCCAATGCCTCTGATGCTTTGGACAAAATCCGCTACGAAAGTCTG    174
               ***** ** ** ********.**.** ***** ****** * ** *****  * **:****** *.*****.***.*.** **.**  *

HSP90AA1.1a    ACAGACCCATCCAAGATGGACTCTGGCAAGGACCTGAAGATCGAGGTCATTCCCAACAAGGAGGAGCGCACCCTGACCCTGGTTGACACC    270
HSP90AA1.2a    ACAGACCCAACCAAATTGGATTCTTGCAAGGAGCTGAAGATCGAGGTCACCCCTGACCTGCGCACTCGTACCCTGACCCTGGTTGACACC    270
HSP90AA1.1b    ACAGACCCGACCAAGATGGACTCTGGCAAGGACCTGAAGATCGAGGTCATTCCCAACAAGGAGGAGCGCACCCTGACCCTGGTTGACACC    270
HSP90AA1.2b    ACAGATCCCACCAAATTGGATTCCTGCAAGGACCTAAAGATCGAGGTCACCCCTGACCTGCGCACTCGCACCCTGACCCTGGTTGACACC    270
HSP90AB1a      ACGGACCCCACCAAGCTGGACAACGGCAAGGAACTGAAGATTGACGTCATCCCCAACGTGGAGGAGCGCACCCTGACCCTAATCGACACT    264
HSP90AB1b      ACGGACCCCACCAAGCTGGACAATGGCAAGGAATTGAAGATTGACATCATCCCCAATGTGGAGGAACGCACCCTGACCCTCATCGACACT    264
               **.** ** :****. **** :.  *******  *.***** ** .***  ** .*  :* . .. ** *********** .* *****

HSP90AA1.1a    GGCATCGGCATGACCAAGGCTGACCTGATCAACAACCTGGGAACCATTGCAAAGTCTGGCACCAAGGCCTTCATGGAGGCCCTGCAGGCT    360
HSP90AA1.2a    GGCATCGGCATGACCAAGGCCGACCTGATCAACAACCTGGGAACCATTGCAAAGTCTGGCACCAAGGCCTTCATGGAGGCCCTGCAGGCT    360
HSP90AA1.1b    GGCATCGGCATGACCAAGGCCGACCTGATAAACAACCTGGGAACCATCGCCAAGTCTGGCACCAAGGCCTTCATGGAGGCCCTGCAGGCT    360
HSP90AA1.2b    GGCATCGGCATGACCAAGGCCGACCTAATAAACAACCTGGGAACCATCGCCAAGTCTGGCACCAAGGCCTTCATGGAGGCCCTGCAGGCT    360
HSP90AB1a      GGAATTGGCATGACCAAAGCTGACCTCATCAACAACCTGGGAACCATCGCGAAGTCTGGCACCAAGGCCTTCATGGAGGCCCTGCAGGCT    354
HSP90AB1b      GGAATTGGCATGACCAAAGCTGACCTCATCAACAACCTGGGAACTATTGCCAAGTCTGGCACCAAGGCCTTCATGGAGGCCCTGCAAGCT    354
               **.** ***********.** ***** **.************* ** ********************************.***

HSP90AA1.1a    GGGGCTGACATCTCCATGATTGGGCAGTTCGGTGTGGGTTTCTACTCTGCCTACCTGGTGGCTGAGAGGGTGACCGTCATCACCAAGCAC    450
HSP90AA1.2a    GGAGCTGACATCTCCATGATCGGGCAGTTCGGTGTGGGTTTCTACTCTGCCTACCTGGTGGCTGAGAGGGTGACTGTCATCACCAAGCAC    450
HSP90AA1.1b    GGAGCTGACATCTCTATGATCGGGCAGTTCGGTGTGGGTTTCTACTCCGCCTACCTGGTGGCTGAGAGGGTGACTGTCATCACCAAGCAC    450
HSP90AA1.2b    GGAGCTGACATCTCTATGATCGGGCAGTTCGGTGTGGGTTTCTACTCCGCCTACCTGGTGGCTGAGAGGGTGACTGTCATCACCAAGCAC    450
HSP90AB1a      GGAGCTGACATCTCCATGATTGGGCAGTTTGGTGTGGGTTTCTACTCTGCCTACCTGGTGGCTGAGAGGGTGACTGTCATCACTAAGCAC    444
HSP90AB1b      GGAGCTGACATCTCCATGATTGGGCAGTTCGGTGTGGGATTCTACTCCGCCTACCTGGTGGCAGAAAGAGTGACTGTCATCACTAAGCAC    444
               **.*********** ***** ******** ********:******** **************:**.**.***** ******** ******

HSP90AA1.1a    AACGATGATGAGCAGTACATCTGGGAATCATCCGCTGGCGGATCCTTCACCGTCAAAGTCGACCCGTCAGCTGAGTCTATTGGTCGTGGG    540
HSP90AA1.2a    AATGATGATGAGCAGTACATCTGGGAGTCTGCAGCTGGTGGCTCTTTCACTGTCAAAGTTGACAC---TGGTGAGTCCATTGGCCGTGGC    537
HSP90AA1.1b    AATGATGATGAGCAGTACATCTGGGAATCCTCTGCTGGCGGATCCTTCACTGTCAAAGTCGACACGTCAGCTGAGTCTATTGGTCGTGGC    540
HSP90AA1.2b    AATGATGATGAGCAGTACATCTGGGAGTCTGCAGCTGGTGGCTCCTTCACTGTCAAAGTTGACAC---TGGTGAGTCCATTGGCCGTGGC    537
HSP90AB1a      AACGATGATGAGCAGTACATCTGGGAGTCCTCAGCCGGAGGCTCCTTCACAGTCAAGGTCGACAC---TGGGGAGCCCATGTTGCGTGGA    531
HSP90AB1b      AATGATGACGAGCAGTACATCTGGGAGTCTTCAGCCGGAGGCTCATTCACAGTCAAGGTCGACAG---TGGGGAGCCCATGTTGCGTGGA    531
               ** ***** ****************.**  * ** ** **.** ***** *****.** ***.     :*  *** * **    *****

HSP90AA1.1a    ACCAAGGTGATTCTGTACCTGAAGGATGACCAGACAGAATACTGTGAGGAGAAACGTGTCAAAGAGATCGTGAAGAAGCACTCCCAGTTC    630
HSP90AA1.2a    ACCAGAGTAATACTGCACATGAAGGAGGACCAGTTTGAATACTGTGAGGAGAAGCGCGTCAAGGAGGTTGTGAAGAAGCACTCTCAGTTC    627
HSP90AA1.1b    ACCAAGGTGATTCTGTACCTGAAGGATGACCAGACAGAATATTGTGAGGAGAAACGGGTCAAAGAAATCGTGAAGAAGCACTCCCAGTTC    630
HSP90AA1.2b    ACCAAAGTGATCCTGCACATGAAGGAGGACCAGTTTGAATACTGTGAGGAGAAGCGCGTCAAGGAGGTTGTGAAGAAGCACTCCCAGTTC    627
HSP90AB1a      ACTAAGGTGATTCTGCACATGAAGGAGGACCAGACGGAGTATGTTGAGGAGAAGAGGGTCAAGGAGGTGGTCAAGAAGCACTCTCAGTTC    621
HSP90AB1b      ACTAAGGTAATTCTGCACATGAAGGAGGACCAGACTGAGTATGTTGAGGAGAAGAGGGTCAAGGAGGTGGTCAAGAAGCACTCTCAGTTC    621
               ** *..**.** *** **.******* ******:   **.**   *********..* *****.**..* ** *********** ******

HSP90AA1.1a    ATCGGATACCCCATCACACTCTTTGTGGAGAAGGAGCGTGACAAGGAAGTGAGTGACGATGAGGCAGAGGAGGAAGAGGAGAAGA---AG    717
HSP90AA1.2a    ATTGGCTATCCCATCACACTCTTTGTGGAGAAGTCTAGAGAGAAAGAGGTG---GACCTT------GAGGAGGGAGAAAAGGATGAGGAG    708
HSP90AA1.1b    ATCGGATACCCCATCACACTCTTTGTGGAGAAGGAGCGTGACAAGGAAGTGAGTGATGATGAGGCGGAGGAGGAGGAGAAGGAGAAGAAA    720
HSP90AA1.2b    ATTGGCTACCCCATCACACTCTATGTGGAGAAGTCTAGAGAGAAGGAGGTG---GACCTT------GAGGAGGGAGAAAAGGATGAGGAG    708
HSP90AB1a      ATTGGATATCCCATCACCCTCTTTGTTGAGAAGGAGCGTGAAAAGGAGATCAGTGACGATGAGGCTGAGGAGGAGGAGAAGGCAGAGAAG    711
HSP90AB1b      ATTGGATATCCCATCACACTTTTCGTTGAAAAGGAGCGTGAAAAGGAGATCAGTGATGATGAGGCTGAGGAGAAGGAGGAA------AAG    705
               ** **.** ********.** *: ** **.*** . .*:** **.**..*   **  :*      ******...**..*.       *.

HSP90AA1.1a    GATGGGGAAGAAGCAGAGAAGAGAGAGGTTGACAAACCCGAGATCGAGGACGTAGGCTCAGACGAGGAGGATCACCATGACCATGACCAC    807
HSP90AA1.2a    GCTGATAAAGATTCTGCAGCTGAGGACCAAGATAAGCCCAAGATCGAAGATGTCGGTTCTGATGAGGATGAGGACACC----AAGG----    790
HSP90AA1.1b    GATGGGG-----------AAGGAGAGGAGGACAAACCTGATATTGAGGATGTAGGCTCGGACGAGGAGGATGACCATGATCATGGCCAC    798
HSP90AA1.2b    GCTGATAAAGATGCTGCAGCTGAGGACAAAGACAAGCCCAAGATCGAGGACGTCGGCTCTGATGAGGACGAGGACACC----AAGG----    790
HSP90AB1a      GAGGAGAAAGAG---GAGAAGGAGGCAGAGGACAAGCCCAAGATTGAGGATGTGGGCTCTGATGATGAGGAGGACTCC----AAAG----    790
HSP90AB1b      GCGGA---GGAA---AAGGCGGAGGCTGAGGACAAGCCCAAAATTGAGGATGTGGGCTCGGATGACGAGGAGGATTCC----AAAG----    781
               *. *.               . ...*.  : ** **.** .* ** **.** ** ** ** ** ** ** **  *  .     *:..

HSP90AA1.1a    GACAGTGCATGCGGTGACAAGAAGAAGAAG---AAGAAGAAGATCAAGGAGAAATACATTGACCAGGAGGAGCTGAACAAGACCAAGCCC    894
HSP90AA1.2a    -------------ATTCCAAGAACAAG---AGGAAGAAGAAGGTCAAGGAGAAGTACATTGATGCAGAAGAGCTGAATAAGACCAAGCCT    864
HSP90AA1.1b    GACGGCGCATGTGGGGACAAGAAGAAGAAGAAGACGAAGAAGATCAAGGAGAAGTACATTGACCAGGAGGAGCTGAACAAGACCAAGCCC    888
HSP90AA1.2b    -------------ACAGCAAGAACAAG---AGGAAGAAGAAGGTCAAGGAGAAGTACATCGACGCAGAGGAGCTGAACAAGACCAAGCCT    864
HSP90AB1a      -------------ACA---AGGACAAGAAAAAGACCAAGAAGATCAAGGAGAAGTACATTGACCAGGAGGAGCTGAACAAGACCAAGCCC    864
HSP90AB1b      -------------ACA---AGGACAAGAAGAAGACTAAGAAGATAAAGGAGAAGTACATTGACCAGGAGGAGCTGAATAAGACCAAGCCC    855
                            .    **.* ***      *. ******.*.********.***** **   ..**.******** ***********

HSP90AA1.1a    CTGTGGACCCGTAACCCCGATGACATCACCAACGAGGAGTATGGAGAGTTCTACAAGAGTCTGACCAACGACTGGGAGGAACACCTGGCT    984
HSP90AA1.2a    ATCTGGACCCGTAACCCTGATGACATCACCAATGAGGAGTACGGAGAGTTCTACAAGAGTCTGACCAACGACTGGGAGGACCACCTGGCT    954
HSP90AA1.1b    CTTTGGACTCGTAACCCTGATGACATCACCAACGAGGAGTACGGAGAGTTCTACAAGAGCCTGACCAACGACTGGGAGGAACACCTGGCT    978
HSP90AA1.2b    ATCTGGACCCGTAACCCTGATGACATCACCAATGAGGAGTACGGAGAGTTCTACAAGAGTCTGACCAACGACTGGGAGGACCACCTGGCT    954
HSP90AB1a      ATTTGGACCAGAAACCCTGACGACATCACCATGGAGGAGTACGGAGAGTTCTACAAGAGCCTGACCAACGACTGGGAGGAACACCTGGCT    954
HSP90AB1b      ATCTGGACCAGGAACCCTGATGACATCACCATGGAGGAGTATGGGGAGTTCTACAAGAGCCTGACCAATGACTGGGAGGACCACCTTGCA    945
               .* ***** .* ***** ** **********: ******** **.************** ******** ***********.***** **:
```

```
HSP90AA1.1a    GTCAAGCACTTCTCAGTTGAGGGCCAGCTGGAGTTCCGTGCCCTGCTCTTTGTGCCTCGCCGTGCACCCTTTGACCTCTTTGAAAACAAG   1074
HSP90AA1.2a    ATCAAGCATTTCTCAGTGGAGGGCCAGCTGGAGTTCCGCGCTCTGCTTTTTGTGCCAAGGAGGGCTTCCTTCGACCTCTTCGAGAACAAG   1044
HSP90AA1.1b    GTCAAGCACTTCTCAGTGGAGGGCCAGCTGGAGTTCCGTGCCCTGCTCTTTGTGCCTCGCCGTGCACCCTTTGACCTCTTTGAGAACAAG   1068
HSP90AA1.2b    ATCAAGCACTTCTCAGTGGAGGGCCAGCTGGAGTTCCGCGCTCTGCTCTTTGTGCCCAGGAGGGCTTCCTTTGACCTCTTTGAGAACAAG   1044
HSP90AB1a      GTCAAGCACTTTTCAGTGGAGGGCCAGCTGGAGTTCCGTGCTCTCCTCTTTATCCCCCGCCGCGCACCCTTCGACCTCTTTGAGAACAAG   1044
HSP90AB1b      GTCAAGCACTTCTCAGTGGAGGGCCAGCTGGAGTTCCGTGCCCTGCTCTTTATCCCCCGCCGCGCACCCTTTGACCTCTTTGAGAACAAG   1035
               .******* ** ***** ******************* ** ** ** ***.* ** .* .* **: **** ******** **.******

HSP90AA1.1a    AAGAAGAAGAACAATATCAAGCTGTACGTCAGGAGGGTCTTCATCATGGACAACTGTGATGATCTTATCCCTGAGTACCTCAACTTCATC   1164
HSP90AA1.2a    AAGAAGAAGAACAACATCAAGCTGTACGTGCGCAGGGTGTTCATCATGGACAACTGTGACGAGCTGATGCCAGAGTATCTCAACTTCATT   1134
HSP90AA1.1b    AAGAAGAAGAACAACATCAAGCTGTACGTCAGGAGGGTCTTCATCATGGACAACTGTGACGATCTGATCCCTGAGTACCTCAACTTCATC   1158
HSP90AA1.2b    AAGAAGAAGAACAACATCAAGCTGTATGTGCGCAGGGTCTTCATCATGGATAACTGTGACGAGCTGATGCCAGAGTATCTCAACTTCATC   1134
HSP90AB1a      AAGAAGAAGAACAACATCAAGCTGTATGTCAGGAGGGTCTTCATCATGGACAGCTGTGAGGAGCTCATCCCGGAGTACCTGAATTTTGTG   1134
HSP90AB1b      AAGAAGAAGAACAACATCAAGCTGTATGTCAGGAGGGTCTTCATCATGGACAGCTGCGAAGAGCTTATCCCAGAGTACCTGAACTTTGTG   1125
               ************* *********** ** .* ***** *********** *.*** ** ** ** ** ** ***** ** ** ** .*

HSP90AA1.1a    AAGGGTGTGGTGGACTCTGAGGATCTCCCCCTAAACATCTCCAGAGAGATGCTGCAGCAGAGCAAGATCCTCAAGGTGATCCGCAAGAAC   1254
HSP90AA1.2a    AAGGGTGTGGTGGACTCTGAGGATCTCCCCCTGAACATCTCCAGAGAGATGCTGCAGCAGAGCAAGATCCTCAAGGTGATCCGCAAGAAC   1224
HSP90AA1.1b    AAGGGTGTGGTGGACTCTGAGGATCTCCCCCTGAACATCTCCAGAGAGATGCTGCAGCAGAGCAAGATCCTCAAGGTGATCCGCAAGAAC   1248
HSP90AA1.2b    AAGGGTGTGGTGGACTCTGAGGATCTCCCCCTGAACATCTCCAGAGAGATGCTGCAGCAGAGCAAGATCCTCAAGGTGATCCGCAAGAAC   1224
HSP90AB1a      CGTGGTGTGGTAGACTCTGAGGATCTCCCCCTGAACATCTCCCGAGAGATGCTGCAGCAGAGCAAGATCCTCAAGGTGATCCGCAAGAAC   1224
HSP90AB1b      CGTGGTGTGGTGGACTCTGAGGATCTCCCCCTGAACATCTCCCGAGAGATGCTGCAACAGAGCAAGATCCTCAAGGTCATCCGCAAGAAC   1215
               .. ********.*******************.*********.*************.****************** ***********

HSP90AA1.1a    CTGGTCAAGAAGTGTATAGAGCTCTTCACTGAGCTGTCAGAGGACAAAGAAAACTACAAGAAGTACTACGAGCAGTTCTCCAAGAACATC   1344
HSP90AA1.2a    CTGGTCAAAAAGTGTATGGATCTTTTCGTCGAGCTCTCAGAAGACAAGGACAACTACAAGAAGTTCTATGAGCAGTTCTCCAAGAACATC   1314
HSP90AA1.1b    CTGGTCAAGAAGTGTATAGAGCTTTTCACAGAACTCTCAGAGGACAGAGATAACTACAAGAAGTACTACGAGCAGTTCTCCAAGAACATC   1338
HSP90AA1.2b    CTGGTCAAGAAGTGTATAGAGCTTTTCATCGAGCTCTCAGAGGACAAGGACAATTACAAGAAGTTCTACGAGCAGTTCTCTAAGAACATC   1314
HSP90AB1a      ATCGTCAAGAAGTGTATGGAGCTGTTCGGCGAGCTGGCAGAGGACAGGGAGAACTACAACAAGTTCTATGATGGCTTCTCCAAGAACCTC   1314
HSP90AB1b      ATCGTCAAGAAGTGTATGGAGCTGTTCGGTGAGCTGGCAGAGGACAAGGAGAACTACAAGAAGTTCTACGATGGCTTCTCCAAGAACCTC   1305
               .* *****.********.** ** ***.  **.**  ****.****..** ** ***** ****:*** **  . ***** ******.**

HSP90AA1.1a    AAGCTGGGGATCCATGAGGACTCTCAGAACCGTAAGAGGCTGTCAGACATGCTGCGCTACTACTCCTCAGCCTC---AGGGGACGAGATG   1431
HSP90AA1.2a    AAGCTGGGAATCCATGAAGATGCTCAGAACCGCAAGAAGCTGTCAGACATGCTGCGCTACTACACCTCCAACTCCAACGCTGACGAAATG   1404
HSP90AA1.1b    AAGCTGGGTATCCATGAGGACTCTCAGAACCGTAAGAGGCTGTCAGACATGCTGCGCTACTACACCTCAGCCTC---AGGGGACGAGATG   1425
HSP90AA1.2b    AAGCTGGGAATCCATGAGGACTCTCAGAACCGCAAGAAATTGTCAGACATGCTGCGCTACTACACCTCCAACTCCGG---TGATGAAATG   1401
HSP90AB1a      AAGCTGGGCATCCACGAGGACTCCCAGAACCGCAAGAAGCTGTCGGAGCTGCTGCGCTACCACAGCTCTCAGTCG---GGAGACGAGCTG   1401
HSP90AB1b      AAGCTGGGGATCCACGAGGACTCCCAAAACCGCAAGAAGCTGTCCGAGCTGCTTCGCTACCACAGCTCCCAGTCT---GGAGATGAGCTG   1392
               ******** ***** **.**  * **.***** ****.. **** ** .**** ****** **: ***  . **      ** **..**

HSP90AA1.1a    GTATCCCTCAAAGACTATGTCACACGCATGAAGGAAACCCAGAAACATATCTACTACATCACTGGCGAGACCAGAGACCAGGTGGCTAAC   1521
HSP90AA1.2a    GTCTCCCTGAAGGAGTATGTTTCTCGCATGAAGGACACCCAGAAACACATCTACTACATAACTGGTGAGACCAAGGAACAGGTCGCCAAC   1494
HSP90AA1.1b    GTCTCCCTAAAGGACTACGTCACACGCATGAAGGACACCCAGAAACACATCTACTACATCACTGGCGAGACCAAAGACCAGGTGGCCAAC   1515
HSP90AA1.2b    GTTTCCTTGAAGGACTACGTTTCCCGCATGAAGGACACCCAGAAACACATCTACTACATTACTGGTGAGACGAAGGAACAGGTCGCCAAC   1491
HSP90AB1a      ACCTCCCTCACGGAGTACCTCACCCGCATGAAGGACAACCAGAAATCCATCTATTACATCACTGGTGAGAGCAAGGACCAGGTGGCCAAC   1491
HSP90AB1b      ACCTCCCTCACAGAGTACCTCACCCGCATGAAGGACAACCAAAAATCCATCTACTACATAACCGGTGAGAGCAAGGACCAGGTGGCCAAC   1482
               .  *** * *..** **  * :* ***********.*.***.*** . ***** ***** ** ** ****  *..**.***** ** ***

HSP90AA1.1a    TCTGCATTTGTGGAACGCCTTCGAAAGGCCGGCCTGGAAGTAATCTACATGATTGAGCCTATTGATGAGTACTGTGTCCAGCAGCTGAAG   1611
HSP90AA1.2a    TCTTCCTTTGTAGAGCGCCTCCGCAAGGCCGGCTTGGAAGTAATCTACATGATTGAACCCATTGATGAGTACTGTGTCCAGCAGCTGAAG   1584
HSP90AA1.1b    TCCGCATTCGTGGAGCGCCTTCGCAAGGCTGGCCTGGAGGTGATCTACATGATTGAGCCCATTGATGAGTACTGTGTCCAGCAGCTGAAG   1605
HSP90AA1.2b    TCTTCCTTTGTGGAGCGCCTCCGCAAGGCCGGCCTGGAAGTGATCTACATGATTGAGCCCATTGATGAGTACTGTGTCCAGCAGCTGAAG   1581
HSP90AB1a      TCTGCTTTTGTGGAGCGCGTGCGCAAGCGAGGATTCGAGGTCCTGTACATGACGGAGCCCATTGACGAGTACTGTGTCCAGCAGCTGAAG   1581
HSP90AB1b      TCAGCCTTTGTAGAACGCGTGCGTAAGCGTGGCTTCGAGGTCCTGTACATGACGGAGCCCATCGATGAATACTGCGTCCAGCAGCTGAAG   1572
               **  * ** **.**.*** * ** ***   **. * **.** .* *******  **.** ** ** **.***** **************

HSP90AA1.1a    GAGTATGATGGCAAGACCCTTGTCTCTGTGACCAAGGAGGGTCTGGAGCTGCCTGAGGACGAGGACATGAAGAAGAGGCATGAAGAACAG   1701
HSP90AA1.2a    GAGTATGATGGCAAGAACCTGGTCTCTGTGACCAAGGAGGGTCTGGAGCTGCCTGAGGATGAGGATGAGAAGAAGAAGCAGGAGGAGCTG   1674
HSP90AA1.1b    GAGTACGATGGCAAGAACCTGGTCTCTGTGACCAAGGAGGGTCTGGAGCTGCCTGAGGATGAAGACATGAAGAAGAGACAAGAAGAGCAG   1695
HSP90AA1.2b    GAGTATGATGGCAAGAACCTAGTCTCTGTGACCAAGGAGGGTCTGGAGCTGCCTGAGGATGAGGATGAGAAAAAGAAACAGGAGGAGCTG   1671
HSP90AB1a      GAGTTTGACGGAAAGACCCTGGTCTCCGTGACCAAGGAGGGCCTGGAGCTGCCGGAGGACGAGGAGGAGAAGAAGAAGATGGATGAGGAC   1671
HSP90AB1b      GAGTTTGACGGTAAAACCCTGGTCTCTGTAACCAAGGAGGGCTTGGAGCTGCCTGAGGATGAGGAGGAGAAGAAGAAGATGGAGGAGGAC   1662
               ****: ** ** **.*.*** ***** **.***********  ********** ***** **.** .:***.****...: ** **. :

HSP90AA1.1a    AAGTCTCAGTTTGAGAACCTCTGCAAGATCATGAAGGACATCTTGGAGAAGAAAGTGGAGAAGGTGACAGTGTCCAACCGCCTGGTCTCC   1791
HSP90AA1.2a    AACACTAAATTCGAGAACCTCTGCAAGACCATGAAGGACATCCTGGACAAGAAGATTGAGAAGGTTTCAGTTTCCAACCGCCTGGTCTCC   1764
HSP90AA1.1b    AAGTCTCAGTTTGAGAACCTCTGCAAGATCATGAAGGACATCCTGGAGAAGAAAGTTGAGAAGGTGACAGTGTCCAACCGCCTGGTCTCC   1785
HSP90AA1.2b    AATTCTAAATTTGAGAACCTTTGCAAGATCATGAAGGACATCCTGGACAAGAAAATTGAGAAGGTTTCAGTGTCCAACCGCCTGGTCTCC   1761
HSP90AB1a      AAGACAAAGTTTGAGAACCTCTGCAAGCTCATGAAGGAGATCCTGGACAAGAAAGTAGAGAAGGTGACCGTGTCAAACAGGCTGGTGTCG   1761
HSP90AB1b      AAGACGAGGTTTGAGAACCTCTGCAAGCTCATGAAGGAGATCCTGGACAAGAAAGTAGAGAAGGTGACTGTGTCAAACAGGCTGGTGTCG   1752
               ** :* ...** ******** ******. ********* *** **** *****..* ******** :* ** **.***.* ***** **

HSP90AA1.1a    TCCCCCTGCTGCATTGTGACCAGCACCTATGGCTGGACGGCCAACATGGAGAGGATCATGAAGGCCCAGGCCCTGAGGGACAACTCCACC   1881
HSP90AA1.2a    TCCCCCTGTTGCATCGTCACCAGTACATACGGCTGGACGGCCAACATGGAGAGAATCATGAAATCTCAAGCTCTCAGAGACAACTCCACC   1854
HSP90AA1.1b    TCCCCCTGCTGCATCGTGACCAGCACCTACGGCTGGACGGCCAACATGGAGAGGATCATGAAGGCCCAGGCCCTGAGGGACAACTCCACC   1875
HSP90AA1.2b    TCCCCCTGCTGCATTGTGACCAGCACCTACGGGTGGACAGCCAACATGGAGAGGATCATGAAATCTCAAGCTCTCAGAGACAACTCCACC   1851
HSP90AB1a      TCGCCCTGCTGCATTGTGACCAGCACGTATGGCTGGACGGCAAACATGGAGAGGATCATGAAGGCGCAGGCCCTGAGGGACAACTCCACC   1851
HSP90AB1b      TCGCCCTGCTGCATCGTGACCAGCACGTATGGCTGGACGGCCAACATGGAGCGCATCATGAAGGCACAGGCCCTGAGGGACAACTCCACA   1842
               ** ***** ***** ** ***** ** ** ** *****.**.*********.* ********. * **.** ** **.***********.

HSP90AA1.1a    ATGGGCTACATGGCTGCCAAGAAGCACCTGGAGATCAACCCAGACCACCCCATTGTGGAGACCCTGAGGCAGAAGGCAGAGGCTGATAAG   1971
HSP90AA1.2a    ATGGGCTACATGACAGCCAAGAAGCACCTGGAGATCAACCCAACCCACCCTATTGTCGAGACTTTGAGAGAGAAAGCTGAAGCTGACAAG   1944
HSP90AA1.1b    ATGGGCTACATGGCTGCCAAGAAACACCTGGAGATCAACCCAGACCACCCCATTGTGGAGACCCTGAGGCAGAAGGCAGAGGCTGATAAG   1965
HSP90AA1.2b    ATGGGCTACATGACAGCCAAAAAGCACCTGGAGATCAACCCAACCCACCCTATTGTCGAGACTCTGAGAGAGAAAGCTGAGGCCGACAAG   1941
HSP90AB1a      ATGGGCTACATGATGGCCAAGAAACACCTGGAGATCAACCCAGACCACCCCATCGTGGAGACCCTGCGGCAGAAGGCTGACCTGGACAAG   1941
HSP90AB1b      ATGGGCTACATGATGGCCAAGAAGCACCTGGAGATTAACCCAGACCACCCCATTGTGGAGACCCTAAGGCAGAAGGCTGACCTGGACAAA   1932
               ************.  *****.**.*********** ******..****** ** ** *****  *..*. ****.**:**    ** **.
```

```
HSP90AA1.1a      AATGATAAATCAGTGAAGGACCTAGTCCTTCTGCTGTTTGAGACGGCTCTGTTGTCCTCTGGGTTCACCTTGGATGACCCTCAGACACAC   2061
HSP90AA1.2a      AACGACAAAGCCGTAAAGGACTTGGTCATCTTGCTGTTCGAGACTGCTCTATTGTCATCTGGGTTCACGCTGGACGACCCTCAGACCCAT   2034
HSP90AA1.1b      AATGATAAGTCTGTGAAGGACCTGGTCATTCTGCTGTTTGAGACGGCTCTACTGTCCTCTGGGTTCACCTTGGATGACCCTCAGACACAC   2055
HSP90AA1.2b      AACGACAAAGCCGTGAAGGACCTGGTCATCTTGCTATTCGAGACTGCTCTGATGTCTTCTGGATTCACACTGGACGACCCTCAGACCCAC   2031
HSP90AB1a        AACGACAAGGCGGTGAAGGACCTGGTAATCCTGCTTTTCGAGACCGCTCTGCTCTCCTCTGGCTTCAGCCTGGATGACCCTCAAACTCAC   2031
HSP90AB1b        AACGACAAGGCGGTGAAGGACTTAGTTATCCTATTATTCGAGACTGCATTGCTCTCTTCGGGCTTCAGCCTGGATGACCCCCAGACTCAC   2022
                 ** ** **. * **.****** *.** .*  *. * ** ***** **: *. * ** ** ** ****   **** ***** **.** **

HSP90AA1.1a      TCCAACCGCATCTACAGAATGATCAAGCTAGGACTGGGTATTGATGAGGATGACCTGACCCCCGAGGAGCC-AACCTCAGCCC-------   2143
HSP90AA1.2a      GCAAACCGCATTTACAGGATGATTAAGCTTGGCCTGGGCATCGATGGTGATGACTCA---GCTGTGGAGGAAATCCT-----CCAGCCCA   2116
HSP90AA1.1b      TCCAACCGAATCTACCGGATGATAAAGCTAGGCCTGGGTATTGACGAGGATGAGCTGACCCCTGAGGAGCC-AACTTCAGCCC-------   2137
HSP90AA1.2b      GCAAATCGCATCTACAGGATGATCAAGCTTGGCCTGGGCATCGATGATGATGATTCA---GCAGTGGAGGACATCCT-----CCAGCCCA   2113
HSP90AB1a        TCCAACCGTATCTACAGGATGATCAAGCTGGGCCTGGGAATCGATGATGACGAAGTGATCCCTGAGGAGC-CCACCTCCGCACCTGCCCC   2120
HSP90AB1b        TCCAACCGCATCTACAGGATGATCAAGCTGGGTCTGGGAATCGATGATGACGAAGTGATCCCCGAGGAAC-CCACCTCTGCACCCGCCCC   2111
                  *.** ** ** ***.*.***** ***** ** ***** ** ** *. ** **    .     * *:***.    .:* *     *

HSP90AA1.1a      CTGTGGAGGACATGCCTCCCCTGGAGGGAGACGAGGACACATCCAGGATGGAGGAGGTTGACTAG 2208
HSP90AA1.2a      GTGAGGATGACATGCCTGTCTTGGAGGGAGATGATGACACATCAAGAATGGAGGAAGTTGACTAA 2181
HSP90AA1.1b      CCATCGAGGACATGCCTCAACTGGAGGGAGATGAGGATACATCCAGGATGGAGGAGGTTGACTAG 2202
HSP90AA1.2b      GTGAGGATGACATGCCTGTCCTGGAGGGAGATGATGACACCTCTAGAATGGAGGAAGTTGACTAA 2178
HSP90AB1a        A----GATGAGATCCCACCTCTAGAGGGAGACGATGATGCATCACGCATGGAGGAAGTGGATTAA 2181
HSP90AB1b        A----GATGAGATCCCACCTCTAGAGGGAGATGAGGATGCTTCACGCATGGAGGAAGTGGATTAA 2172
                     ** ** ** **:     *.******** ** ** .* ** .* ********.** ** **.
```

Figure S28: Nucleotide alignment of Chinook salmon HSP90AA1.1a (GenBank accession no. OP760297), HSP90AA1.1b (GenBank accession no. OP760298), HSP90AA1.2a (GenBank accession no. OP760296), HSP90AB1.2b (GenBank accession no. OQ215311), HSP90AB1a (GenBank accession no. OP760299) and HSP90AB1b (GenBank accession no. OP760300) cDNA sequences. Exons are highlighted with a different colour. Identical (*) nucleotides identified by the Clustal omega program are indicated. Positions where the forward and reverse primers are found are boxed.

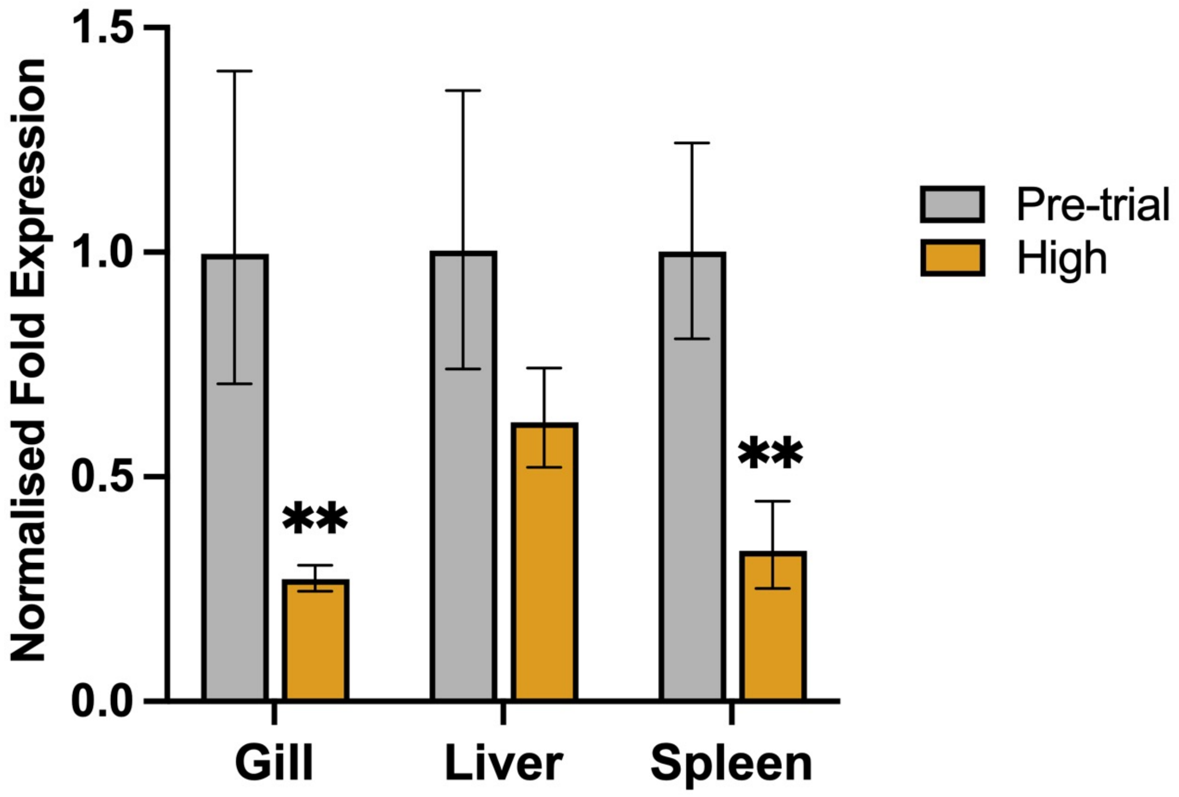

Figure S29: Normalised fold expression of SOD1 in the liver, spleen and gill of pre-trial control fish (Pre-trial) and fish kept at high-temperature/low-DO (High). Error bars represent the standard error. ** indicates p<0.02 and * indicates p<0.05.

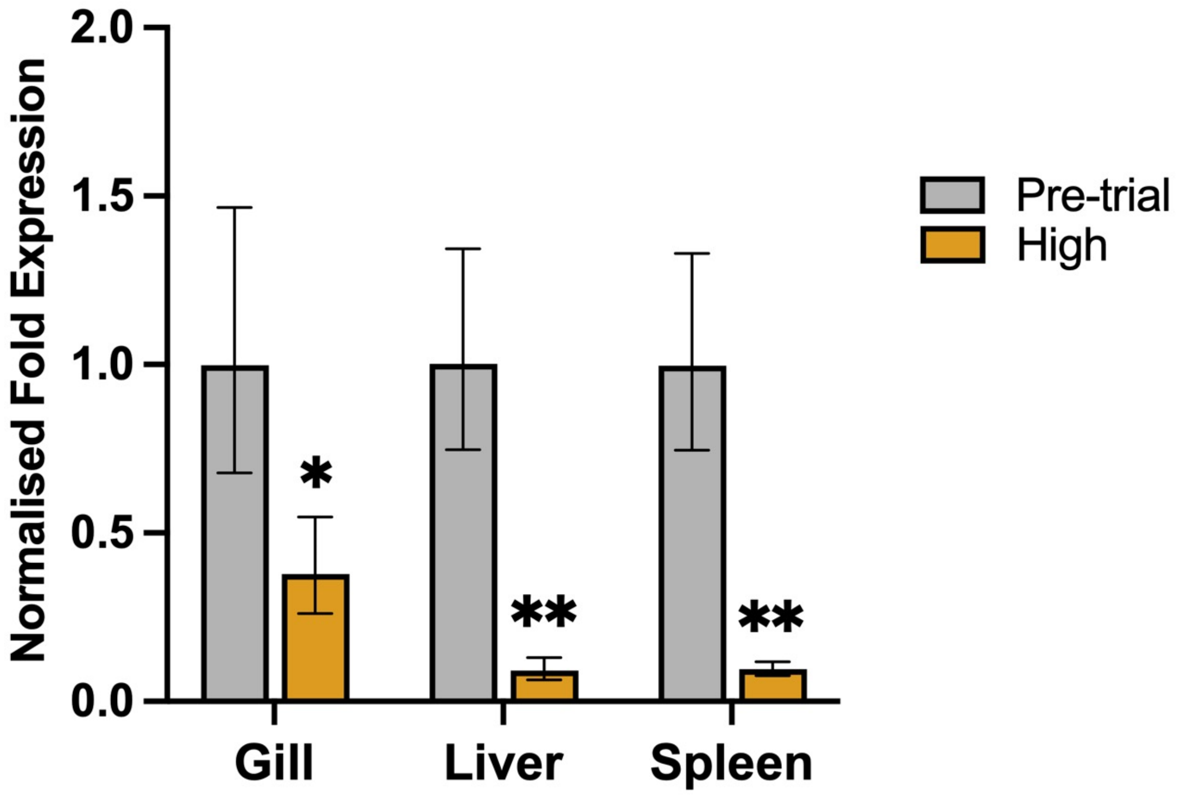

Figure S30: Normalised fold expression of HSP90 in the liver, spleen and gill of pre-trial control fish (Pre-trial) and fish kept at high-temperature/low-DO (High). Error bars represent the standard error. ** indicates p<0.02 and * indicates p<0.05.
