# Supplementary material for: Characterising the Physiological Responses of Chinook Salmon (Oncorhynchus tshawytscha) Subjected to Heat and Oxygen Stress
Source: Biology (Basel). 2023 Oct 17;12(10):1342. doi: 10.3390/biology12101342 (PMC10604766; doi:10.3390/biology12101342)
Supplement: Supplementary file 1 [file biology-12-01342-s001.zip › biology-2497437-supplementary/Supplementary figure 10 update.pptx]

## Slide 1
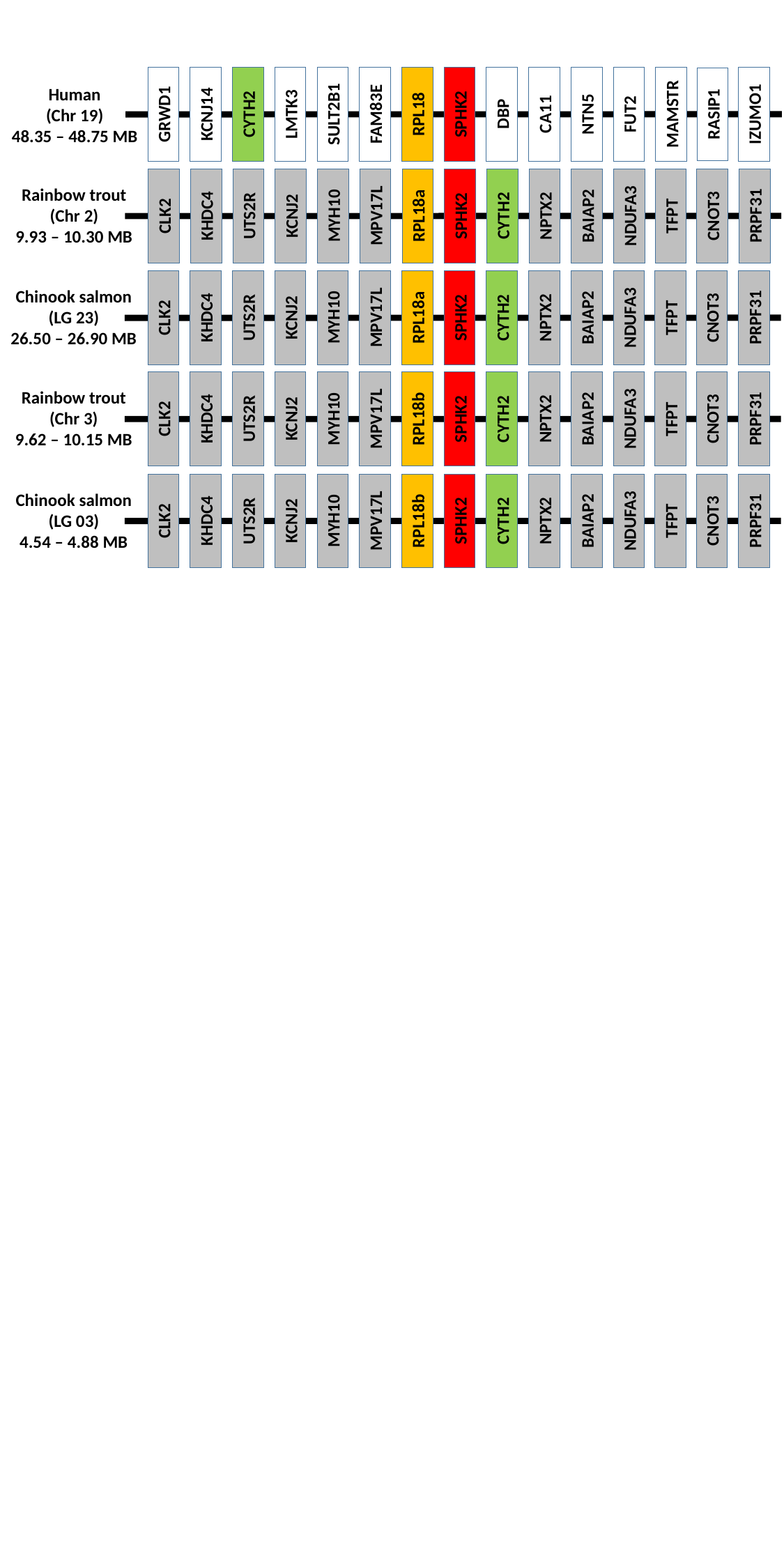

Human
(Chr 19)
48.35 – 48.75 MB
GRWD1
KCNJ14
CYTH2
LMTK3
SULT2B1
FAM83E
RPL18
SPHK2
DBP
CA11
NTN5
FUT2
MAMSTR
RASIP1
IZUMO1
Rainbow trout
(Chr 2)
9.93 – 10.30 MB
PRPF31
CLK2
KHDC4
UTS2R
KCNJ2
MYH10
MPV17L
RPL18a
SPHK2
CYTH2
NPTX2
BAIAP2
NDUFA3
TFPT
CNOT3
Chinook salmon
(LG 23)
26.50 – 26.90 MB
PRPF31
CLK2
KHDC4
UTS2R
KCNJ2
MYH10
MPV17L
RPL18a
SPHK2
CYTH2
NPTX2
BAIAP2
NDUFA3
TFPT
CNOT3
Rainbow trout
(Chr 3)
9.62 – 10.15 MB
PRPF31
CLK2
KHDC4
UTS2R
KCNJ2
MYH10
MPV17L
RPL18b
SPHK2
CYTH2
NPTX2
BAIAP2
NDUFA3
TFPT
CNOT3
Chinook salmon
(LG 03)
4.54 – 4.88 MB
PRPF31
CLK2
KHDC4
UTS2R
KCNJ2
MYH10
MPV17L
RPL18b
SPHK2
CYTH2
NPTX2
BAIAP2
NDUFA3
TFPT
CNOT3
