# Supplementary material for: Characterising the Physiological Responses of Chinook Salmon (Oncorhynchus tshawytscha) Subjected to Heat and Oxygen Stress
Source: Biology (Basel). 2023 Oct 17;12(10):1342. doi: 10.3390/biology12101342 (PMC10604766; doi:10.3390/biology12101342)
Supplement: Supplementary file 1 [file biology-12-01342-s001.zip › biology-2497437-supplementary/Supplementary figure 16 update.pptx]

## Slide 1
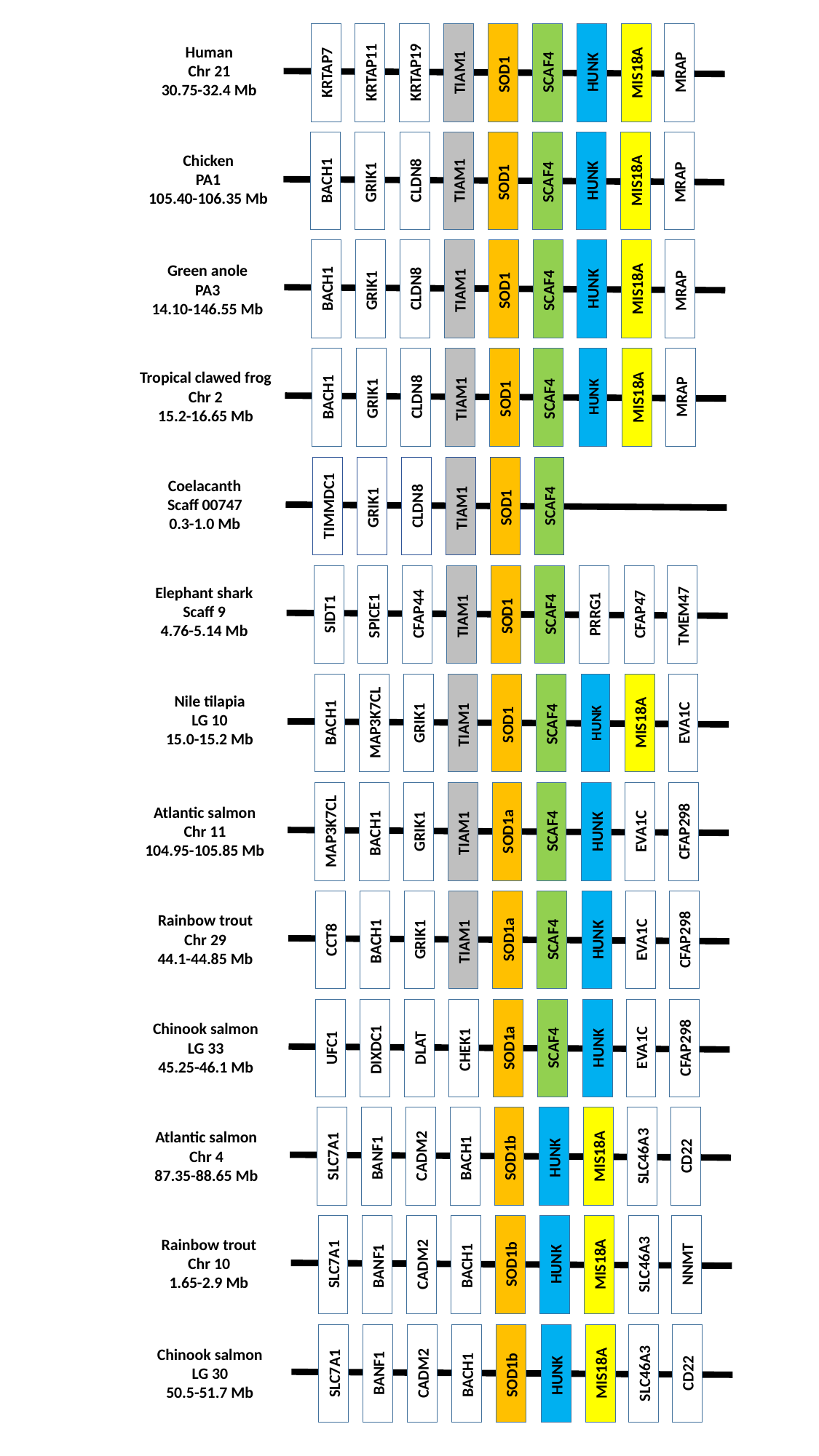

Human
Chr 21
30.75-32.4 Mb
KRTAP11
KRTAP19
TIAM1
SOD1
SCAF4
HUNK
MRAP
KRTAP7
MIS18A
Chicken
PA1
105.40-106.35 Mb
GRIK1
CLDN8
TIAM1
SOD1
SCAF4
HUNK
MRAP
BACH1
MIS18A
Green anole
PA3
14.10-146.55 Mb
GRIK1
CLDN8
TIAM1
SOD1
SCAF4
HUNK
MRAP
BACH1
MIS18A
Tropical clawed frog
Chr 2
15.2-16.65 Mb
GRIK1
CLDN8
TIAM1
SOD1
MRAP
BACH1
SCAF4
MIS18A
HUNK
Coelacanth
Scaff 00747
0.3-1.0 Mb
GRIK1
CLDN8
TIAM1
SOD1
TIMMDC1
SCAF4
Elephant shark
Scaff 9
4.76-5.14 Mb
SPICE1
CFAP44
TIAM1
SOD1
SCAF4
PRRG1
TMEM47
SIDT1
CFAP47
Nile tilapia
LG 10
15.0-15.2 Mb
MAP3K7CL
GRIK1
TIAM1
SOD1
EVA1C
BACH1
SCAF4
MIS18A
HUNK
Atlantic salmon
Chr 11
104.95-105.85 Mb
BACH1
GRIK1
TIAM1
SOD1a
CFAP298
MAP3K7CL
SCAF4
HUNK
EVA1C
Rainbow trout
Chr 29
44.1-44.85 Mb
BACH1
GRIK1
TIAM1
SOD1a
CFAP298
CCT8
SCAF4
HUNK
EVA1C
Chinook salmon
LG 33
45.25-46.1 Mb
DIXDC1
DLAT
CHEK1
SOD1a
CFAP298
UFC1
SCAF4
HUNK
EVA1C
Atlantic salmon
Chr 4
87.35-88.65 Mb
BANF1
CADM2
BACH1
SOD1b
CD22
SLC7A1
HUNK
MIS18A
SLC46A3
Rainbow trout
Chr 10
1.65-2.9 Mb
BANF1
CADM2
BACH1
SOD1b
NNMT
SLC7A1
HUNK
MIS18A
SLC46A3
Chinook salmon
LG 30
50.5-51.7 Mb
BANF1
CADM2
BACH1
SOD1b
CD22
SLC7A1
HUNK
MIS18A
SLC46A3
