# Supplementary material for: Characterising the Physiological Responses of Chinook Salmon (Oncorhynchus tshawytscha) Subjected to Heat and Oxygen Stress
Source: Biology (Basel). 2023 Oct 17;12(10):1342. doi: 10.3390/biology12101342 (PMC10604766; doi:10.3390/biology12101342)
Supplement: Supplementary file 1 [file biology-12-01342-s001.zip › biology-2497437-supplementary/Supplementary figure 4 update.pptx]

## Slide 1
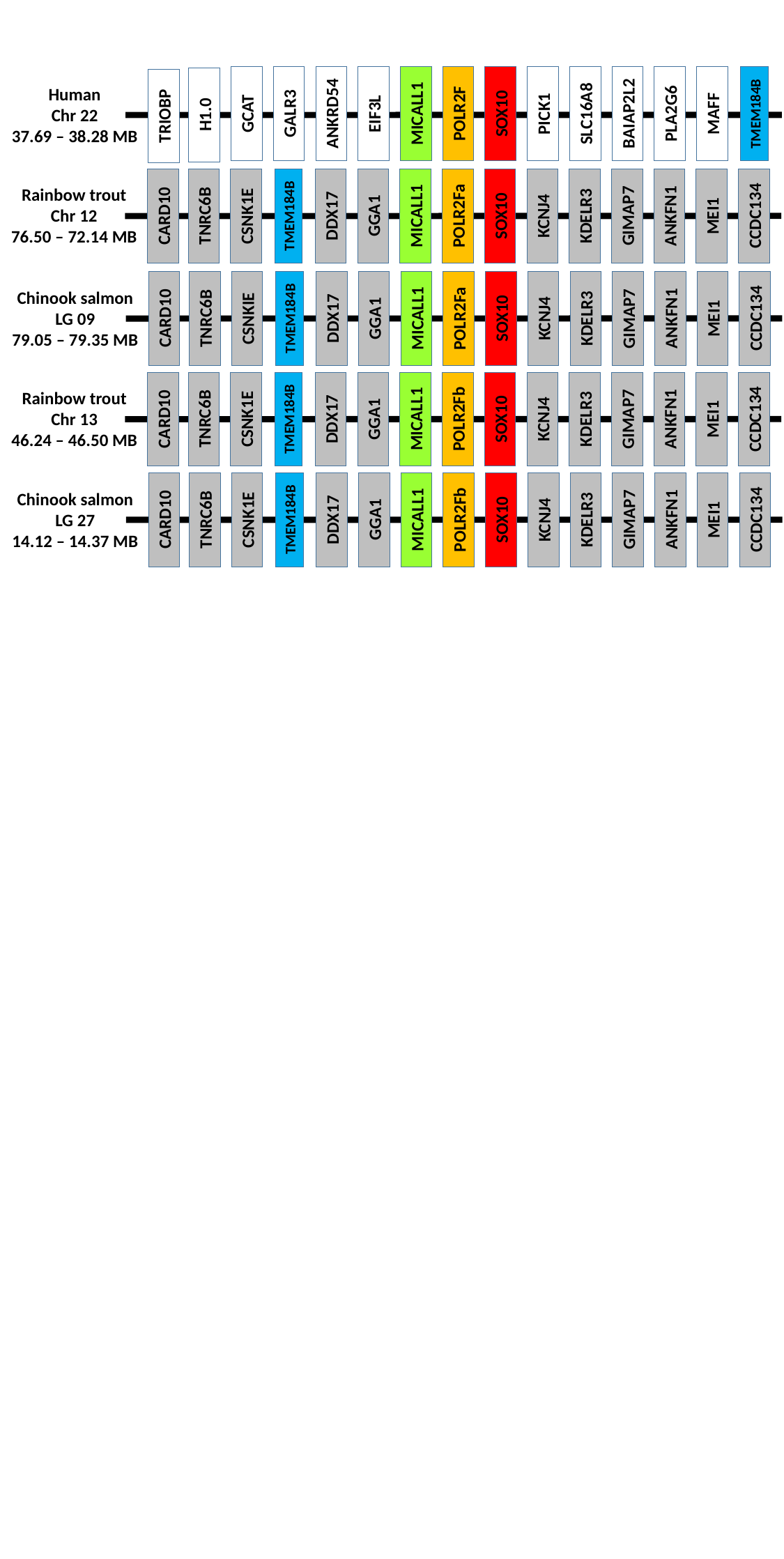

Human
Chr 22
37.69 – 38.28 MB
BAIAP2L2
POLR2F
GCAT
GALR3
ANKRD54
EIF3L
MICALL1
SOX10
PICK1
SLC16A8
PLA2G6
MAFF
H1.0
TMEM184B
TRIOBP
Rainbow trout
Chr 12
76.50 – 72.14 MB
CARD10
TNRC6B
CSNK1E
DDX17
GGA1
MICALL1
POLR2Fa
SOX10
KCNJ4
KDELR3
GIMAP7
ANKFN1
MEI1
CCDC134
TMEM184B
Chinook salmon
LG 09
79.05 – 79.35 MB
CARD10
TNRC6B
CSNKIE
DDX17
GGA1
MICALL1
POLR2Fa
SOX10
KCNJ4
KDELR3
GIMAP7
ANKFN1
MEI1
CCDC134
TMEM184B
Rainbow trout
Chr 13
46.24 – 46.50 MB
CARD10
TNRC6B
CSNK1E
DDX17
GGA1
MICALL1
POLR2Fb
SOX10
KCNJ4
KDELR3
GIMAP7
ANKFN1
MEI1
CCDC134
TMEM184B
Chinook salmon
LG 27
14.12 – 14.37 MB
CARD10
TNRC6B
CSNK1E
DDX17
GGA1
MICALL1
POLR2Fb
SOX10
KCNJ4
KDELR3
GIMAP7
ANKFN1
MEI1
CCDC134
TMEM184B
