# Supplementary figures and images for: Characterising the Physiological Responses of Chinook Salmon (Oncorhynchus tshawytscha) Subjected to Heat and Oxygen Stress
Source: Biology (Basel). 2023 Oct 17;12(10):1342. doi: 10.3390/biology12101342 (PMC10604766; doi:10.3390/biology12101342)

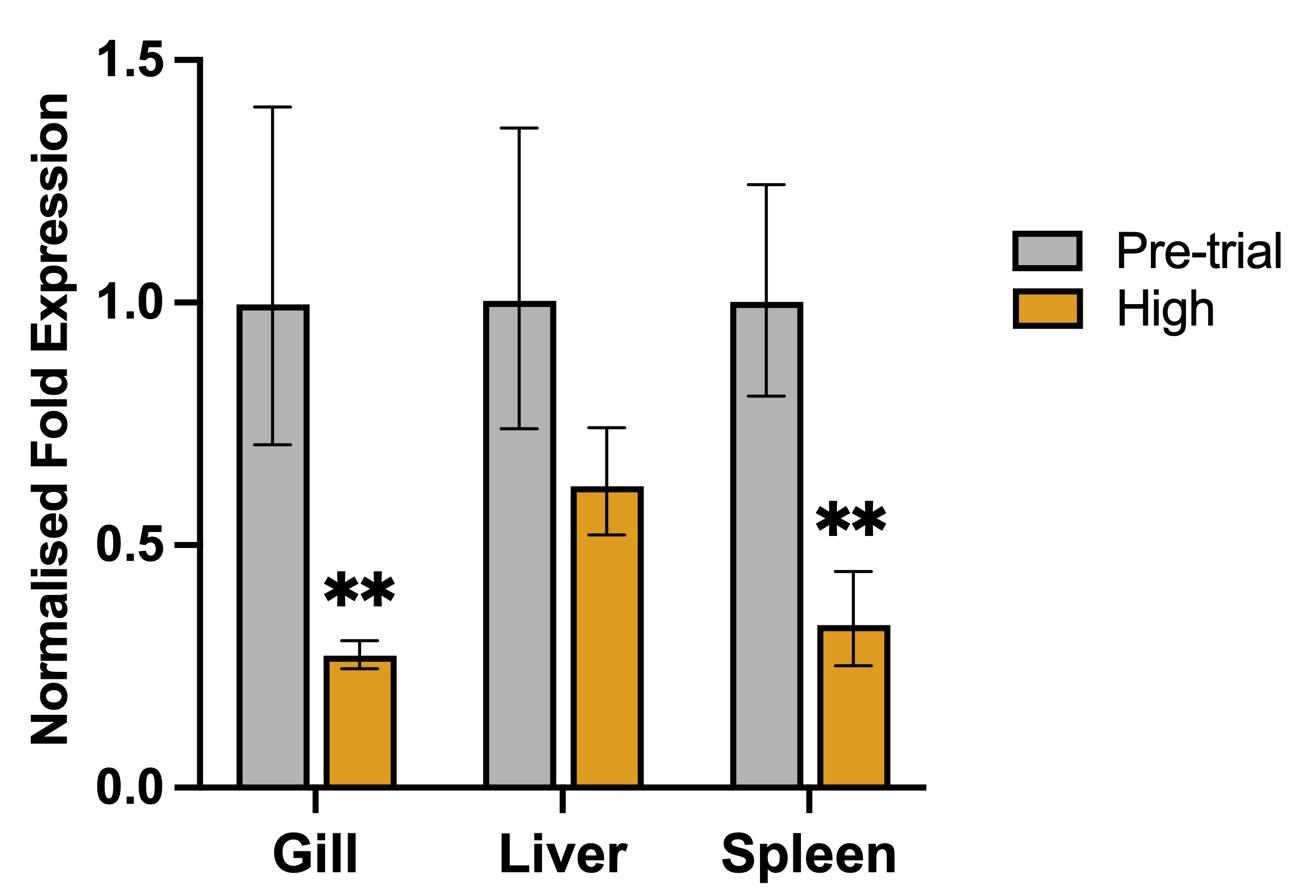

Supplement: Supplementary file 1 [file biology-12-01342-s001.zip › biology-2497437-supplementary/Supplementary figure 29 update.jpg]

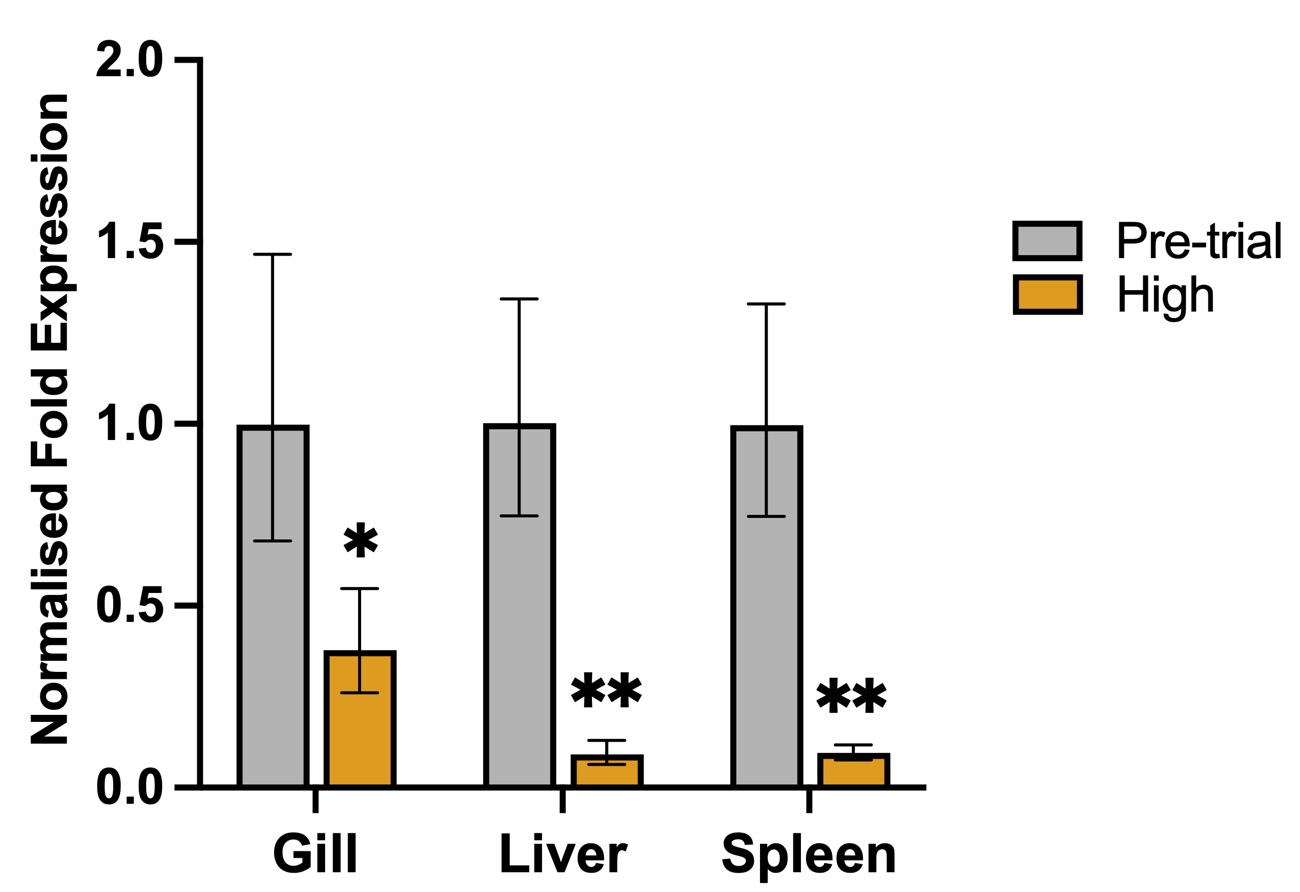

Supplement: Supplementary file 1 [file biology-12-01342-s001.zip › biology-2497437-supplementary/Supplementary figure 30 update.jpg]
